# Molecular cloning, expression, and characterization of acyltransferase from *Pseudomonas protegens*

**DOI:** 10.1007/s00253-018-9052-z

**Published:** 2018-05-12

**Authors:** Nina G. Schmidt, Anna Żądło-Dobrowolska, Valerie Ruppert, Christian Höflehner, Birgit Wiltschi, Wolfgang Kroutil

**Affiliations:** 1 ACIB GmbH, Graz, Austria; 2 Institute of Chemistry, University of Graz, NAWI Graz, BioTechMed Graz, Graz, Austria

**Keywords:** Acyltransferase, 2,4-diacetylphloroglucinol, *Pseudomonas protegens*, Friedel-Crafts reaction

## Abstract

The formation of C-C bonds by using CoA independent acyltransferases may have significant impact for novel methods for biotechnology. We report the identification of *Pseudomonas* strains with CoA-independent acyltransferase activity as well as the heterologous expression of the enzyme in *E. coli*. The cloning strategies and selected expression studies are discussed. The recombinant acyltransferases were characterized with regard to thermal and storage stability, pH,- and co-solvent tolerance. Moreover, the impact of bivalent metals, inhibitors, and other additives was tested. Careful selection of expression and working conditions led to obtain recombinant acyltransferase form *Pseudomonas protegens* with up to 11 U mL^−1^ activity.

**Electronic supplementary material:**

The online version of this article (10.1007/s00253-018-9052-z) contains supplementary material, which is available to authorized users.

## Introduction

**Fig. 1:**
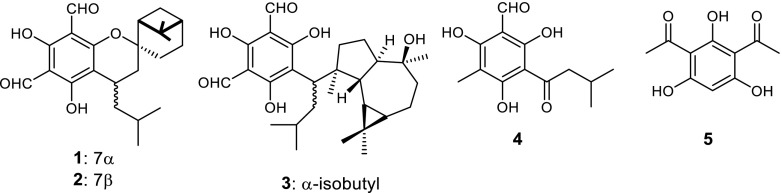
Biologically active phloroglucinol derivatives

The phenolic compound phloroglucinol (PG, 1,3,5-trihydoxybenzene) is a polyketide and represents the key scaffold of around 700 natural products isolated from different natural sources including microorganisms, plants, and marine (Singh and Bharate 2006, Singh et al. 2010). This includes halogenated, prenylated or acylated phloroglucinols, phloroglucinol glycosides, and cyclic polyketides or terpene-adducts. Those natural compounds exhibit a broad spectrum of antibacterial, antiviral, antifungal, antihelminthic, phytotoxic, and antioxidant properties (Tada et al. 1990; Bae 2011; Mathekga et al. 2000; Artan et al. 2008; Veena et al. 2016). For instance, robustadials A and B are known for their in vivo antimalarial activity (**1**–**2**, Fig. 1; Xu et al. 1984), macrocarpal B possesses potent HIV-RTase inhibitory activity (**3**, Fig. 1; Nishizawa et al. 1992), and gerandinol (**4**, Fig. 1) shows Epstein-Barr virus inhibitory activity (Takasaki et al. 1990).

Among acylated phloroglucinols, the bis-acetylated compound 2,4-diacetylphloroglucinol (**5**; DAPG, Fig. 1) is of special interests due to its strong antibiotic properties. DAPG is a secondary metabolite excreted by several plant-associated *Pseudomonas* sp. and *Pseudomonas fluorescens* ssp*.* to protect the rhizosphere from soil-borne pathogens (Dowling and O'Gara 1994). The biosynthesis of DAPG is regulated by the *phlACBDEFGHI* gene cluster, conserved among all DAPG-producing *Pseudomonas* strains (Keel et al. 1996; Moynihan et al. 2009), which is divided into regulatory genes *phlEFGHI* and the biosynthetic operon *phlACBD*.

In addition to complex genetic regulations, several biotic and abiotic factors were found to modulate DAPG production as well (Notz et al. 2001; Matano et al. 2010; Paulin et al. 2009). Among the proteins which are not directly involved in the biosynthesis, *phlE* encodes a putative permease (Abbas et al. 2004) and *phlG* a hydrolase (Bottiglieri and Keel 2006; Saitou et al. 2012). *PhlF* and *phlH* are transcriptional regulators (Schnider-Keel et al. 2000) and *phlI* is a so-far uncharacterized protein. Among the biosynthetic genes, *phlD* encodes a type-III polyketide synthase which is responsible for the biosynthesis of the DAPG-precursor phloroglucinol (PG) (Achkar et al. 2005; Zha et al. 2006; Cao and Xian 2011; Yang and Cao 2012). Finally, the operon *phlACB* encodes an acetyl-CoA independent acyltransferase (ATase), which catalyzes the acetylation of PG leading to the target polyketide DAPG (Shanahan et al. 1993; Bangera and Thomashow 1999). Expression of the entire *phlACB* operon is essential to obtain functional ATase, since individual expression of *phlA*, *phlC*, and *phlB* and subsequent incubation of the three individual proteins did not show any activity towards disproportionation of the natural substrate monoacylphloroglucinol (MAPG) (Bangera and Thomashow 1999; Achkar et al. 2005; Hayashi et al. 2012). This leads to the assumption that the ATase exists as a multienzyme complex, what was furthermore confirmed by the fact that mutations in any of the genes resulted in a loss of catalytic activity (Bangera and Thomashow 1999; Kidarsa et al. 2011). According to literature, biocatalytic applications were limited to *phlD* which was employed for the in-vivo production of PG in either *E. coli* or *Pseudomonas* sp. under controlled conditions in bioreactors (Cao et al. 2011; Banotai et al. 2012; Frost 2012; Rao et al. 2013).

**Fig. 2:**
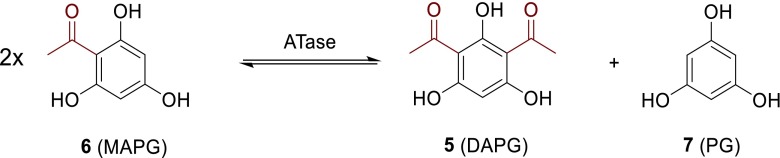
Natural reaction catalyzed by the acyltransferase (ATase) involved in the biosynthesis of DAPG

Very recently, a multi-component acyltransferase (ATase) originating from the bacterium *Pseudomonas* sp. YGJ3 was identified to catalyze the reversible disproportionation of two molecules of MAPG (**6**, Fig. 2) into one molecule of PG (**7**) and DAPG (**5**) in the forward reaction (Hayashi et al. 2012; Yang and Cao 2012; Almario et al. 2017). Our previous report proved that a multi-component ATase from *Pseudomonas protegens* catalyzes transfer of acyl moieties, not only from natural but also from non-natural donor substrates, to the aromatic ring of a phenolic acceptor substrate by forming a new C-C bond in a Friedel-Crafts-type acylation reaction (Schmidt et al. 2017). In this work, we report on the cloning of the key biosynthetic operon *phlACB*, into a bacterial expression vector to produce the ATase in *E. coli* and developed optimal conditions for robust synthesis of this enzyme.

## Materials and methods

### General information

All starting materials in this study were obtained from commercial suppliers (Sigma- Aldrich, Alfa Aesar, or TCI-Chemicals) and used as received unless stated otherwise. 1,3-Diacetyl-2,4,6-trihydroxy benzene (**5**, DAPG) was chemically synthesized as previously described (Sato et al. 2005; Khazaei et al. 2010; Mudududdla et al. 2012). pH-measurements were carried out on a pH Meter (Hanna Instruments, HI2211 pH/ORP Meter), equipped with a conventional Ag/AgCl pH-electrode (SI-Analytics, BlueLine 16 pH). *Pseudomonas protegens* DSM 19095 and *Pseudomonas brassicacearum* DSM 13227 were obtained from the Deutsche Sammlung von Mikroorganismen und Zellkulturen GmbH (DSMZ). *Pseudomonas fluorescens* Pf-5 was obtained from the American Type Culture Collection (ATCC BAA-477). gBlocks® gene fragments, primers for polymerase-chain reactions (PCR) were purchased from IDT and Eurofins MWG Operon. The expression vector pASK-IBA3plus was purchased from IBA©-Solutions for Lifesciences. Restriction enzymes and the PureLink® Genomic DNA Minikit were obtained from ThermoFisher Scientific. Chemically competent *E. coli* DH5α and *E. coli* BL21 (DE3) cells and the Gibson Assembly® master mix were purchased from New England Biolabs. Conversions for product **9** were determined by HPLC (Shimadzu-prominence liquid chromatograph, SPD-M20A diode array detector), equipped with a Phenomenex Luna® 5 μ C18 (2) 100A (250 × 4.6 mm) column. Gradient elution with H_2_O and MeCN (+TFA, 0.1 vol%) was applied: 0–15% MeCN (0–5 min), 15–60% MeCN (5–22 min), 60–100% MeCN (22–25 min), 100–0% MeCN (25–30 min), flow rate = 1 mL min^−1^, sample vol. = 2 μL, λ = 280 nm. GC-MS spectra were recorded with an Agilent 7890A GC-system (Agilent 5975C mass selective detector) equipped with a HP-5 MS column (30 m × 0.25 mm × 0.25 μm); injector 250 °C, constant flow 0.7 mL; carrier gas = He. Temperature program 100 °C (hold 0.5 min) 100 to 300 °C (10 °C min^−1^), 300 °C (hold 2 min). ^1^H- and ^13^C-NMR spectra were recorded at 20 °C on a 300 Bruker NMR unit; chemical shifts are given in ppm relative to Me_4_Si (^1^H: Me_4_Si = 0.0 ppm) or relative to the resonance of the solvent (^1^H: acetone-*d6* = 2.05 ppm; ^13^C: acetone-*d6* = 29.84 and 206.6 ppm). TLC was carried out with pre-coated aluminum sheets (TLC Silica gel 60 F_254_, Merck) with detection by UV (254 nm) and/or by staining with cinnamaldehyde/HCl solution [abs. EtOH (72.2 vol%), conc. HCl (3.6 vol%), *trans*-cinnamaldehyde (3.6 vol%)]. Shake flask cultivation, expression of the wild-type, and recombinant ATase was performed as described previously (Schmidt et al. 2017).

### Shake flask cultivation of the Pseudomonas wildtypes

For the cultivation of the *Pseudomonas* wildtypes, *P. protegens* and *P. brassicacearum*, a pre-culture (100 mL) containing M1-media (peptone 5 g L^−1^, meat-extract 3 g L^−1^) was inoculated with a glycerol stock (250 μL). The pre-culture was grown at 28 °C (*P. protegens*) or 30 °C (*P. brassicacearum*) for 3 days and shaken with 120 rpm. The main culture (3 × 330 mL) in baffled shake flasks (1 L) was inoculated with the pre-culture (10 mL), and incubation was continued for another 3 days under the given conditions. The cells were harvested by centrifugation (10 min, 8000 rpm) and washed with potassium phosphate buffer (50 mM, pH 7.5) prior to lyophilization.

### Plasmid construction

The *phlACB* operon from *P. protegens* DSM19095 and *P*. *brassicacearum* DSM13227 was amplified from the genomic DNA using primer sequences which were identified in a BLAST-search (Supporting Information). The *phlACB* open-reading frames (≈ 2770 bp) of *P*. *protegens* DSM19095 and *P*. *brassicacearum* DSM13227 were identified from a multiple sequence alignment using the *phl* gene cluster sequence *phlACBDEFGHI* of *Pseudomonas* sp. YGJ3 as template (Noyori 2009). Since *P. protegens* DSM19095 shares 99% sequence identity to the reference strain *Pseudomonas* sp. YGJ3, primer sequences for the amplification of *phlACB* were taken from literature without further modifications (Hayashi et al. 2012). Whereas, *P. brassicacearum* DSM13227 was unavailable in databases, *P. brassicacearum* NFM241 was chosen as a best substitute with 80% sequence identity. A second sequence alignment indicated 99% identity between *phl* genes of *P. brassicacearum* NFM241 and *P. fluorescens* J2. For this reason, the *phlACB* genes of *P. fluorescens* J2 were used to design the primers for *P. brassicacearum* DSM13227. The isolated WT-ATase encoding genes were subsequently cloned into pASK-IBA3 (*tet*-promoter) to obtain the plasmids pEG330 and pEG331.

### Construction of recombinant *Pseudomonas protegens*

To construct the recombinant *Pp*ATaseCH, the ATase encoding open-reading frames *phlA*, *phlC*, and *phlB* of *P. protegens* were codon-optimized by manually matching the codon-frequency of the *Pseudomonas* wild-type with *E. coli*. For this purpose, codon-usage tables for *Escherichia coli* B and *Pseudomonas fluorescens* were obtained from the Kazusa-database (http://www.kazusa.or.jp/codon/). Ribosomal binding sites suitable for *E. coli* were introduced upstream of each start codon of every individual *phl* gene. The optimized *phl* genes were obtained as gene fragments (gBlocks©) and assembled together with the double-digested pASKIBA3plus backbone (*Eco*RI/*Hin*dIII) by Gibson cloning (Gibson Assembly® master mix) and subsequent overlap extension-PCR (OE-PCR). The resulting expression vector carries the *E. coli* codon-optimized ATase encoding genes *phlACB* under the control of the P_Tet_ promoter.

### Shake flask cultivation and expression of the recombinant ATases

The expression plasmids (pEG330-*Pb*ATaseWT, pEG331-*Pp*ATaseWT or pEG332-*Pp*ATaseCH) were chemically transformed into *E. coli* BL21 (DE3) host cells. A single colony, picked from an agar-plate (LB/ampicilin, 100 μg mL^−1^), was used for inoculation of an overnight-culture (10 mL, LB/Amp, 100 μg mL^−1^) which was grown at 37 °C and 135 rpm for 15 h. The main culture (1 L) containing LB/Amp (100 μg mL^−1^) was then inoculated with the overnight culture (10 mL) and shaken in a non-baffled flask (5 L) at 37 °C and 140 rpm until the OD_600_ reached 0.7. The cells were induced with anhydrotetracycline (AHTC, 200 μg L^−1^), and protein expression was continued for 21 h at 30 °C (*Pp*ATaseWT or *Pp*ATaseCH) or 6 h at 25 °C (*Pb*ATaseWT). The cells were harvested by centrifugation (15 min, 8000 rpm, 12,028×*g*), washed with potassium phosphate buffer (50 mM, pH 7.5), and resuspended again in the same buffer (7 mL buffer to 1 g wet cells). The suspension was disrupted by ultrasonication (40% amplitude, 8 min, pulse 1 s, pause 4 s). After centrifugation (30 min, 14,000 rpm, 23,519×*g*), the cell-free extract was analyzed by SDS-PAGE, shock-frozen in liquid nitrogen and stored at − 20 °C until direct use for biotransformations. Initial rates and protein concentrations were measured to determine the batch activity.

### Acylation of resorcinol with DAPG - general procedure

Resorcinol (**8**, 1.1 mg, 0.01 mmol, 10 mM final concentration) was dissolved in KPi-buffer (50 mM, pH 7.5), and the mixture was preheated to 35 °C for 10 min. Cell-free *E. coli* extract containing the recombinant ATase (50 μL or vol. ≡ 20 mg lyophilized cells) was added, and the reaction was started by addition of DAPG (3.15 mg, 0.015 mmol, 15 mM final concentration) dissolved in DMSO (100 μL). DMSO was required to improve the solubility of the donor in buffer. The final reaction mixture (1 mL, 10 vol% DMSO) was shaken for 18 h at 35 °C and 550 rpm in an Eppendorf benchtop shaker. The reaction was aborted by addition of HPLC-grade MeCN (1 mL) and vigorous shaking. The precipitated protein was removed by centrifugation (7 min, 14,000 rpm, 18,407×*g*) and the supernatant (800 μL) was transferred to an Eppendorf tube and left standing for another 40 min. Any precipitated protein was removed by centrifugation (10 min, 14,000 rpm, 18,407×*g*), and the supernatant was directly subjected to HPLC for determination of conversions.

### Activity assay

ATase-batch activities were measured on a Thermo Scientific Genesys 10 UV Scanning U*V*/Vis spectrophotometer according to a modified procedure (Hayashi et al. 2012) from literature. When following the disproportionation of MAPG into DAPG and PG spectrophotometrically (Fig. 2), an increase of absorption is recorded due to the formation of DAPG (*ε* = 205.6 M^−1^ cm^−1^, *λ* = 395 nm). One unit of activity was defined as the amount of ATase that catalyzed the formation of 1 μmol of DAPG per minute under the following conditions: potassium phosphate buffer (800 μL, 50 mM, pH 7.5) and MAPG (2.0 μmol, 100 μL of a 20 mM stock solution prepared in DMSO) were added to a cuvette and preheated to 35 °C. The reaction (1 mL total volume, 10 vol% DMSO) was started by the addition of the enzyme-containing cell-free extract (100 μL ≡ 14.3 mg wet cells). The reaction was followed for 3 min. All reactions were performed as a duplicate. As a negative control, a sample with cell-free extract of *E. coli* without plasmid (empty host cells) was run. The protein concentration (Bradford) was measured [*ε* = 0.083 mL mg^−1^ cm^−1^, *λ* = 595 nm], and specific activities were determined as units per mg cell-free extract or as units per mg purified protein. PG does not absorb at this wavelength.

Nucleotide sequences used in this study have been deposited at NCBI.

*Pb*ATaseWT (wild-type nucleotide *phl*ACB gene sequence, *P. brassicacearum* DSM 13227, Genbank accession no.: KY173354).

*Pp*ATaseWT (wild-type nucleotide *phl*ACB gene sequence, *P. protegens* DSM 19095, Genbank accession no.: CP003190.1).

*Pp*ATaseCH (codon-optimized *phl*ACB gene sequence, *P. protegens* DSM 19095, Genbank accession no.: KY173355).

## Results

### Screening of *Pseudomonas* wild type strains

**Fig. 3:**
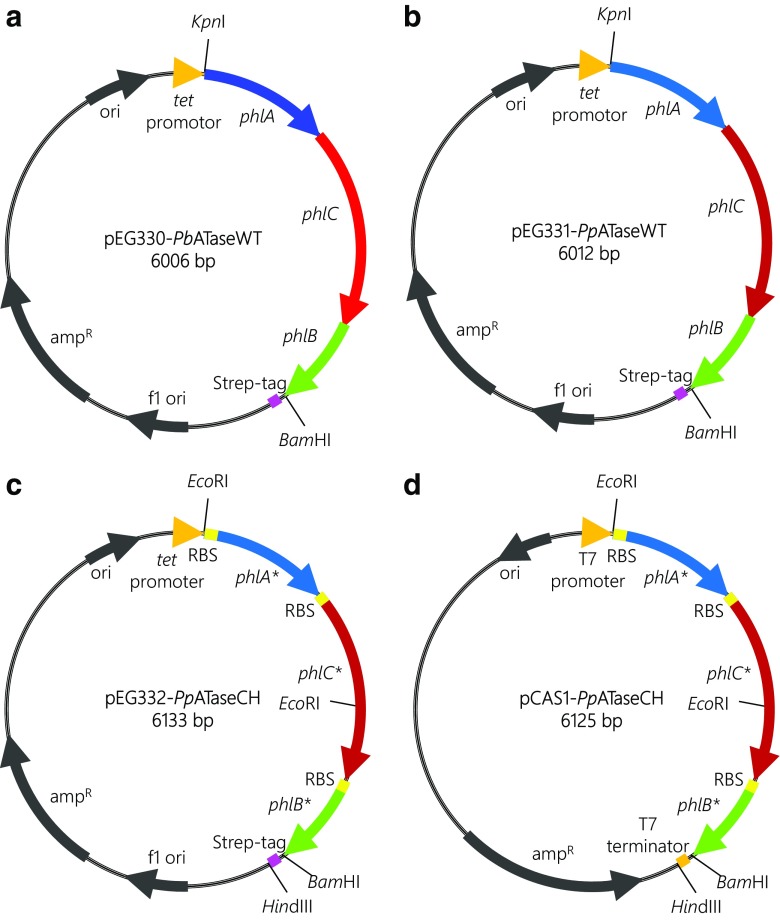
Plasmids harboring **a** the WT-derived ATase genes *phlACB* from *P. brassicacearum* (*Pb*ATaseWT, pEG330), **b** the WT-derived ATase genes *phlACB* from *P. protegens* (*Pp*ATaseWT, pEG330), **c** the codon-harmonized phlACB* genes and ribosmomal binding sites (RBS), optimized ATase from *P. protegens* (*Pp*ATaseCH, pEG332), *tet*-promoter regulated, **d** optimized ATase from *P. protegens* (*Pp*ATaseCH, pCAS1), T7-promoter regulated

Freeze-dried cell preparations of the respective *Pseudomonas* wild-type were screened for ATase activity. For this purpose, 32 strains from the in-house culture collection (Table S1, Fig. S1) were qualitatively assayed for the acetylation and deacetylation of MAPG. Additionally, the reverse direction, the acetylation of PG by DAPG, was tested. Among the investigated *Pseudomonas* wild-type strains, three strains were found to be active, namely *P. brassicacearum* DSM13227, *P. fluorescens* Pf-5 ATCC BAA-477, and *P. protegens* DSM19095. The *phlACB* operon from *P. protegens* DSM19095 and *P. brassicacearum* DSM13227 was amplified from the genomic DNA using primer sequences which were identified in a BLAST-search. The isolated WT-ATase encoding genes were subsequently cloned into pASK-IBA3 (*tet*-promoter) to obtain the plasmids pEG330 (*Pb*ATaseWT, Fig. 3) and pEG331 (*Pp*ATaseWT, Fig. 3b).

### Expression of the wild-type acyltransferases

**Fig. 4:**
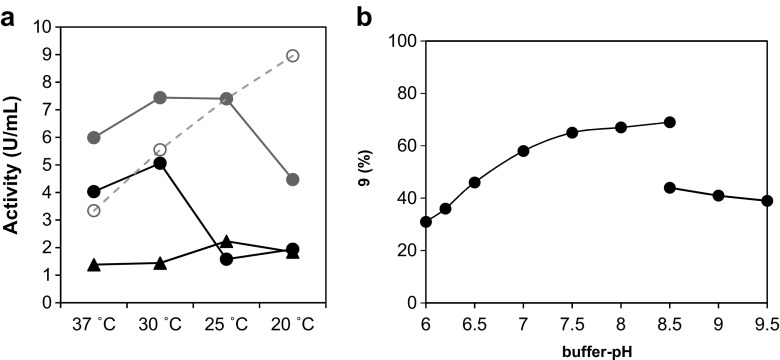
**a** Batch activities (units per mL cell-free extract) of *Pp*ATaseWT (black circles), *Pb*ATaseWT (black triangles) after expression in LB-media for 21 h in the absence of ZnCl_2_, and *Pp*ATaseCH in the presence (open gray circles) and absence of ZnCl_2_ (filled gray circles) at varied temperatures. **b** pH-study of the *Pp*ATaseCH. Assay conditions: Lyophilized cells of *E. coli* containing the recombinant *Pp*ATaseCH (20 mg), KPi-buffer (50 mM) for pH 6.0–8.5 or CHES-buffer (50 mM) for pH 8.5–9.5, resorcinol (**8**, 10 mM), DAPG (15 mM), DMSO (10 vol%), 35 °C, 30 min, 750 rpm

In order to establish the optimum expression conditions required to obtain soluble and active protein, parameters such as temperature, culture media, and time, were investigated. Both WT-ATases were expressed in *E. coli* BL21 (DE3) host cells, and ATase activities were measured spectrophotometrically, following the disproportionation of MAPG into DAPG and PG. The activity of the *Pp*ATaseWT was highest when expression was performed at 30 °C with a rapid decrease in activity below 30 °C, whereas 25 °C was the optimum expression temperature for the *Pb*ATaseWT (Fig. 4a).

**Fig. 5:**
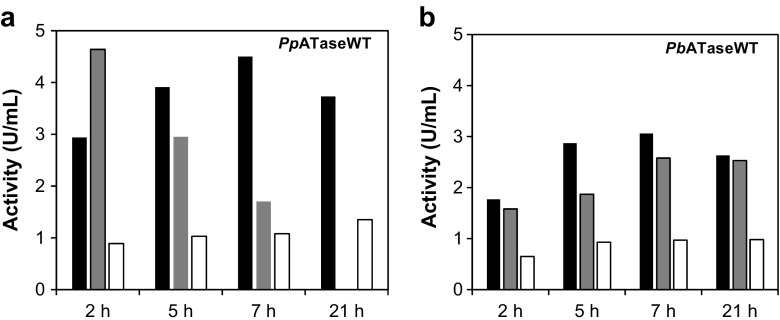
**a** Activities of *Pp*ATaseWT and **b** *Pb*ATaseWT (units per mL cell-free extract) after expression at different times in different culture media: LB-media (black column), TB-media (gray column), YENB-media (white column). The activity of the *Pp*ATaseWT in TB after 21 h was not determined

In the next step, the effect of different growth media on the expression of the WT-ATases was studied at various time frames. LB-, TB-, or YENB-broth media were tested at previously determined temperatures, thus 30 °C for *Pp*ATaseWT and 25 °C for *Pb*ATaseWT. Results are shown in Fig. 5. Activities after expression of the *Pp*ATaseWT in TB-media linearly decreased with increasing expression time. Even though a 2 h expression in TB gave the best results in terms of protein activity, cell densities were too low to provide enough protein after such a short expression time. The use of chloride-free YENB-media suffered from low activities for both enzymes.

### Cloning strategy for *Pp*ATaseCH

To obtain a higher and more balanced expression of the three subunits of the ATase encoding genes in *E. coli*, *phlACB* from *P. protegens* (Almario et al. 2017) were codon-harmonized and ribosomal binding sites (RBS) were introduced at the 5′-end of each *phl* gene. For this purpose, the optimized phl* genes were assembled by Gibson cloning (Smith and March 2007; Turner and O'reilly 2013) and overlap-extension PCR (OExPCR) (Fessner and Anthonsen 2009; Patel 2006; Bommarius and Riebel-Bommarius 2004) to obtain the *tet*-promoter regulated plasmid pEG332 (*Pp*ATaseCH, Fig. 3c). Alternatively, the phl* genes were cloned into the T7-promoter-regulated vector pCAS1 to obtain the construct pCAS1- *Pp*ATaseCH (Fig. 3d). Expression conditions optimized for the related *Pp*ATaseWT served as a starting point for the expression of the codon-harmonized *Pp*ATaseCH (pEG332), i.e., *E. coli* BL21, LB-media, 30 °C, 21 h. The cell-free extracts produced by codon-harmonized construct *Pp*ATaseCH displayed twofold higher activities towards disproportionation of MAPG compared to analogous preparations derived from wild-type sequences. According to SDS-PAGE, all *phl* genes of the codon-harmonized ATase were clearly overexpressed in high amounts and soluble form, in contrast to the WT-ATase, which was not clearly detectable at the same conditions (see Supporting Information). The expression of the *Pp*ATaseCH was performed efficiently under control of the P_*Tet*_ promoter, whereas, the expression under regulation of the strong T7-promoter (pCAS1-construct) did not lead to functional ATase.

### Influence of Zn^2+^ on the expression

Since the impact of Zn^2+^ on the functional expression of the ATase was of particular interest, the influence of ZnCl_2_ (1 mM) and temperature was studied in detail (Fig. 4a). Batch activities determined for *Pp*ATaseWT (Fig. 4a, black circles) and *Pp*ATaseCH (Fig. 4a, open gray circles) after expression with ZnCl_2_ were compared to the expression without additives (Fig. 4a, gray-filled circles). The expression level of *Pp*ATasCH was enhanced in the presence of Zn^2+^ as deduced from the SDS-page (Supporting Information, SDS-gel); however, the obtained activities in the cell-free extract at 30 °C were 1.5 times lower compared to the expression in the absence of ZnCl_2_ (Fig. 4a, filled vs. open gray circles). Even more interesting was the effect of Zn^2+^ at various temperatures. Again, Zn^2+^ led to slightly enhanced visible expression, whereas the activity linearly increased with decreasing temperature, thereby reaching the highest activity at 20 °C.

### Cultivation scale and size of shake flasks

To evaluate the possible influence of the cultivation scale and the size of the shaking flasks on the measured ATase activities, total protein concentration and enzyme specific activity were determined after cultivation in various size of baffled and non-baffled flasks. Although there are variations (Table 1, entry 4) for related conditions, the activity obtained expressed as U/mL was comparable (Entries 1–3).

**Table 1:** Activities depending on the scale of the shake flask cultivation and the flask size

Entry	Flask size (L)	Medium/flask (L)	Total protein (mg/mL)	U/mL	U/mg
1	0.3^[a]^	0.006	20.03	7.44	0.49
2	1.0^[a]^	0.33	15.25	8.90	0.58
3	5.0	1.0	14.85	8.2	0.55
4	5.0	1.0	8.95	10.95	1.22

Cultivation conditions: LB-media, 200 μg L^−1^ AHTC, 30 °C, 21 h, 140 rpm

^[a]^Baffled flask

### pH-study and co-solvent tolerance

**Fig. 6:**
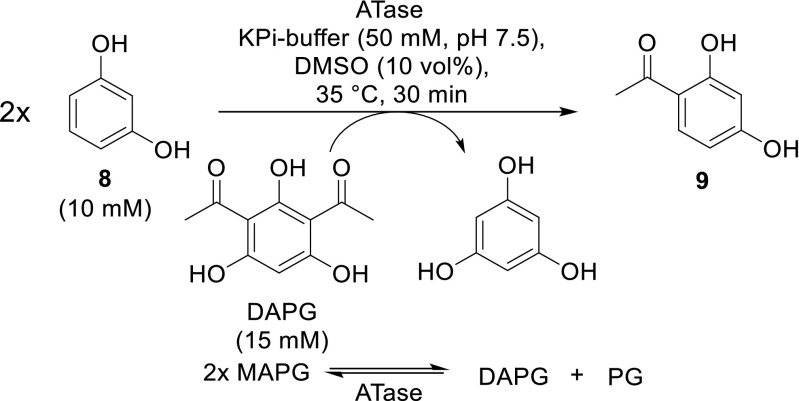
The bioacetylation of resorcinol (**8**) by DAPG served as a new model system to characterize the ATases

For the evaluation of pH, the model substrate was changed to resorcinol (Fig. 6), since we were interested to apply this enzyme for various acyl donors and acceptors (Schmidt et al. 2017). Thus, the ATase activity in the following studies was evaluated based on the formation of the C-acetyl product **9** arising from the bioacetylation of model substrate **8** by DAPG. Alternatively to DAPG, also MAPG may be used as an acyl donor. However, bioacylation of **8** performed with MAPG resulted in 13% product yield within 30 min, while using DAPG gave 65% in the same conditions (KPi-buffer pH 7.5, 35 °C, 750 rpm). Following the disproportionation of MAPG into DAPG and PG on 10 mM scale resulted in 59% conversion.

The pH-optimum was determined based on the ATase catalyzed formation of **9** upon bioacetylation of **8** with DAPG at varied pH-values (Fig. 4b). pH-values, from slightly acidic (pH 6.0) to alkaline (pH 9.5) were investigated using two buffer salts, namely KPi-buffer (50 mM) for pH 6.0–8.5 and CHES-buffer (50 mM) for pH 8.5–9.5. The *Pp*ATaseCH showed the highest activity at slightly alkaline pH, as product formation was most efficient when reactions were carried out between pH 7.5 and 8.5 KPi-buffer.

Besides temperature and pH, the application of co-solvent was considered. Different water-immiscible (toluene, cyclohexane), moderately water-miscible (MTBE, DIPE, Et_2_O, EtOAc), aprotic water-miscible (DMSO, DMF, THF, 1,4-dioxane, acetone, MeCN), and protic water-miscible (MeOH, EtOH, glycerol, ethylene glycole) solvents were tested for the bioacetylation of model substrate **8** either at 5 vol% (Fig. S11, black columns) or 20 vol% (Fig. S11, gray columns), and the compatibility with *Pp*ATaseCH was determined based on the formation of C-acetyl product **9**. It is worth to note that only toluene, cyclohexane, and MTBE formed a biphasic system, whereas the other solvents were completely miscible under the given conditions. Most of the investigated co-solvents were well tolerated by the enzyme at both tested concentrations, although no substantial improvements were observed compared to the reaction without solvent (Fig. S11, gray dashed line). Weakly water-miscible ethers, such as MTBE, DIPE or diethyl ether, and the water-miscible aprotic solvent DMSO, seemed to be slightly preferred, because the formation of **9** went almost equally well at 5 and 20 vol% concentration, respectively. Larger differences with respect to conversion and solvent concentration were obtained with the water-miscible ones. Especially THF and, at higher concentrations, also DMF as well as several other protic and aprotic co-solvents turned out to be rather detrimental to the ATase.

### Storage and thermo-stability of different types of enzyme preparation

**Fig. 7:**
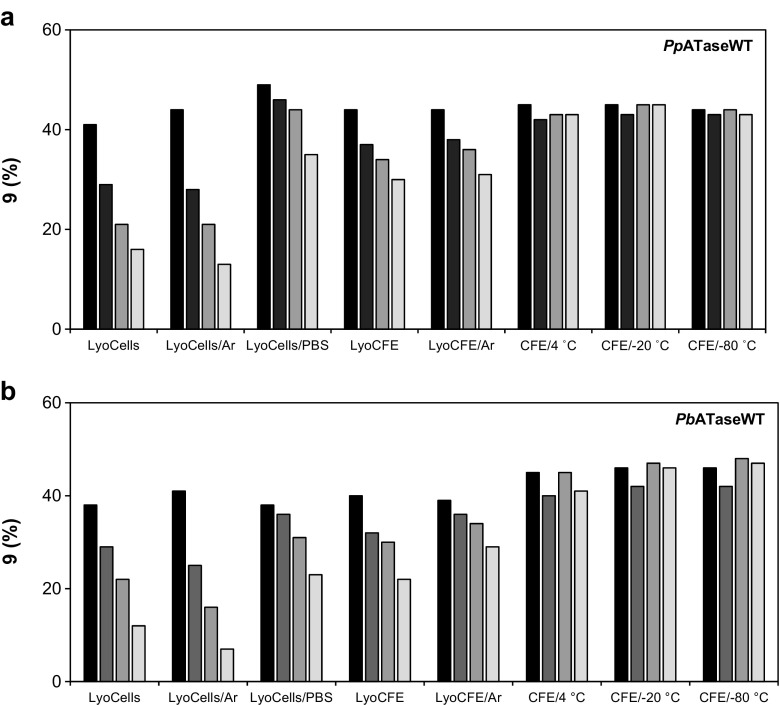
Studies on the storage stability of different types of enzyme preparations **a** *Pp*ATaseWT and **b** *Pb*ATaseWT. The conversions were measured after 0 days (black columns), 14 days (dark gray columns), 35 days (pale gray column), and 63 days (white column) after preparing the catalyst. Assay conditions: Cell preparation of the respective recombinant ATase (20 mg or vol. ≡ 20 mg lyophilisate), KPi-buffer (50 mM, pH 7.5), resorcinol (**8**, 10 mM), DAPG (15 mM), DMSO (10 vol%), 35 °C, 30 min, 750 rpm; LyoCells, (lyophilized cells, KPi-buffer, 4 °C); LyoCells/Ar, (lyophilized cells, inert storage, KPi-buffer, 4 °C); LyoCells/PBS (Lyophilized cells, PBS, 50 Mm, pH 7.5, 4 °C), LyoCFE, (lyophilized cell-free extract, KPi-buffer, 4 °C); LyoCFE/Ar, (lyophilized cell-free extract, inert storage, KPi-buffer, 4 °C); CFE/4 °C, (liquid cell-free extract, KPi-buffer, 4 °C); CFE/− 20 °C, (liquid cell-free extract, KPi-buffer, − 20 °C); CFE/− 80 °C, (liquid cell-free extract, KPi-buffer, − 80 °C)

To learn about the storage stability of the recombinant ATases, batch activities of different types of enzyme preparations under various conditions were monitored over 9 weeks (Fig. 7). To test the residual activity of the enzyme batches, the bioacetylation of **8** with DAPG was performed after 0, 14, 35, or 63 days of storage. The impact of lyophilization and storage temperature was evaluated. Additionally, the sensitivity to molecular oxygen was tested by storing the preparation under inert atmosphere (argon). For instance, freeze-dried cells prepared in KPi-buffer lost almost their entire activity after being stored for 9 weeks at 4 °C; inert storage under argon did not help to retain activity (Fig. 7a and b, rows 1 and 2). However, if cells were treated in salt-rich PBS-buffer instead of KPi-buffer prior to lyophilization, the activity loss was significantly slower, indicating that high salt concentrations contribute to the stabilization of the enzyme (Fig. 7, row 3). If the ATase-containing cell-free extract was lyophilized and stored at 4 °C, activity was lost as well. However, the degradation was much slower than observed for the lyophilized cells (Fig. 7, rows 4–5 vs. rows 1–2). Interestingly, when the liquid cell-free extract was frozen or simply stored at 4 °C instead of lyophilized, the entire batch activity was retained for at least 9 weeks. Preparations stored at + 4 °C, − 20 or − 80 °C were equally active (Fig. 7, rows 6–8) leading to **9** with 44–45% product yield.

**Fig. 8:**
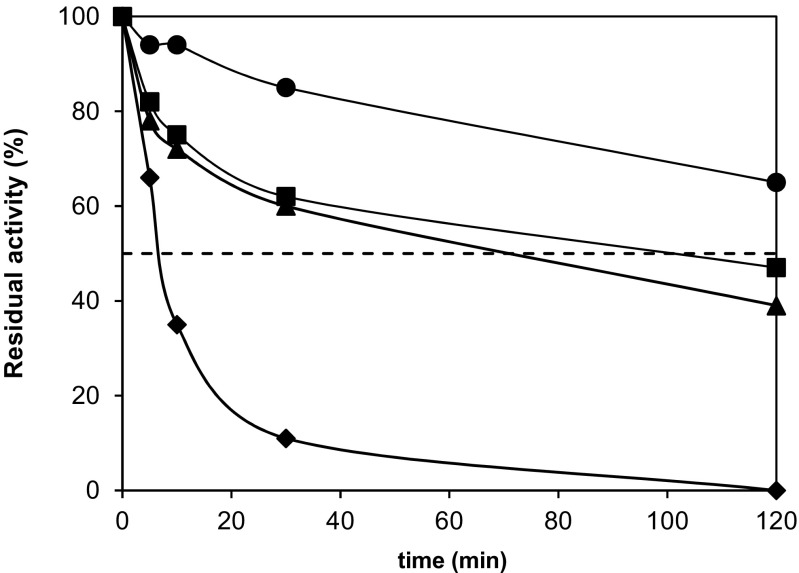
Thermostability of *Pp*ATaseCH. The residual activity was determined after heat-treating the ATase-containing cell-free extract at 35 °C (circle), 50 °C (square), 60 °C (triangle), or 70 °C (diamond) for up to 120 min. Assay conditions: Cell-free *E. coli* extract containing the recombinant *Pp*ATaseCH (50 μL), KPi-buffer (50 mM, pH 7.5), resorcinol (**8**, 10 mM), DAPG (15 mM), DMSO (10 vol%), 35 °C, 30 min, 750 rpm. Activities in % were related to an untreated control sample (= 100%)

Tolerance to an increased reaction temperature is a crucial parameter having significant effects on both, the enzymatic activity and its stability. The thermostability of *Pp*ATaseCH was determined by heat-treatment of the ATase-containing free extract assay at various temperatures. When incubating the ATase at 35 °C, the residual activity after 2 h was 68% (Fig. 8, circle); however, at higher temperatures, such as 50 °C (Fig. 8, square) or 60 °C (Fig. 8, triangle), the enzyme lost half of its activity within 70 min. Prolonging the incubation time at these temperatures up to 2 h resulted in residual activities of approximately 40%. At even higher temperatures, severe heat-deactivation was noticed. For instance, at 70 °C (Fig. 8, diamond) the half-life was less than 10 min and consequently, after 2 h the enzyme was not active anymore.

### Inhibitors and additives

**Fig. 9:**
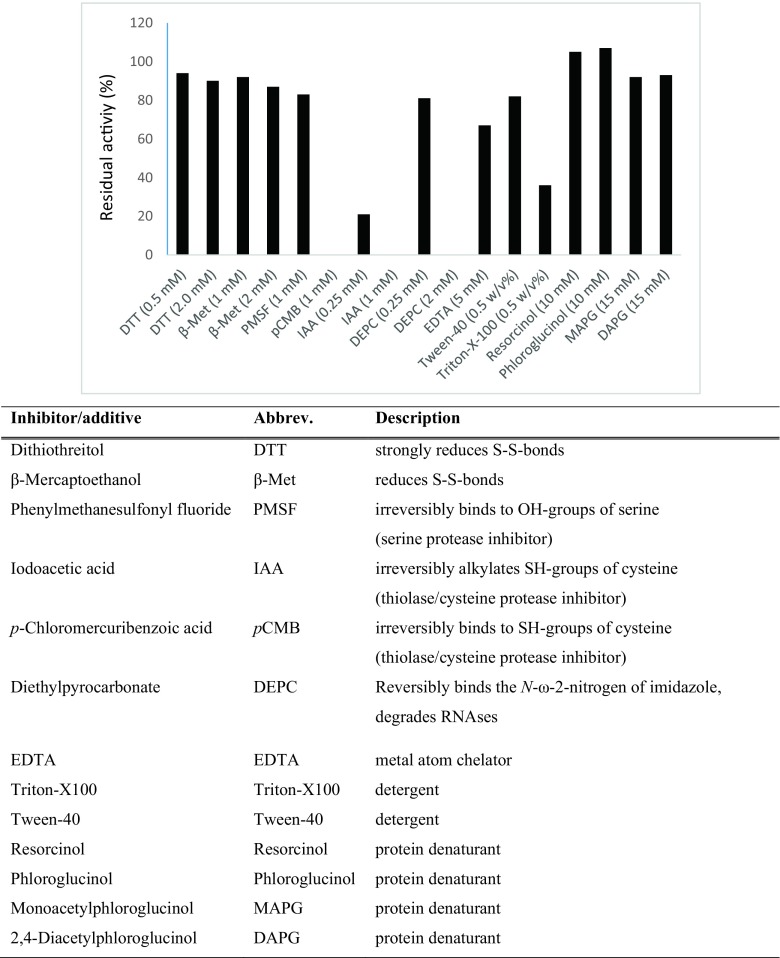
The influence of inhibitors and additives on *Pp*ATaseCH. The residual activity was determined after pre-treating the ATase-containing cell-free extract at 28 °C for 40 min with the respective additive. Assay conditions: Cell-free *E. coli* extract containing the recombinant *Pp*ATaseCH (50 μL), KPi-buffer (50 mM, pH 7.5), resorcinol (**8**, 10 mM), DAPG (15 mM), DMSO (10 vol%), 35 °C, 30 min, 750 rpm. Activities in % were related to an untreated control sample (= 100%)

The influence of commonly used inhibitors was studied in order to gain more information about the ATase (Fig. 9). For testing a small aliquot of cell-free *E. coli* extract containing the recombinant ATase (50 μL) was pretreated with the respective inhibitor/additive for 40 min at 28 °C: dithiothreitol (DTT, 0.5 or 2 mM), 2-mercaptoethanol (β-Met, 1 or 2 mM), phenylmethanesulfonyl fluoride (PMSF, 1 mM), iodoacetic acid (IAA, 1 or 2 mM), *p*-chloromecuribenzoic acid (*p*CMB, 1 mM), diethylpyrocarbonate (DEPC, 2 or 3 mM), EDTA (5 mM), Triton-X100 (0.5 *w*/*v*%), or Tween-40 (0.5 *w*/*v*%). All studied inhibitors affected the ATase activity, leading in general to a loss of activity (Fig. 9). After treatment with *p*CMP or IAA, which are known for their ability to covalently bind to thiol moieties, the enzyme activity was abolished. Furthermore DEPC, which is known to modify histidine residues also led to a loss of conversion at 2 mM concentration. DTT, β-Met, PMSF, and EDTA led to slightly reduced activities. Furthermore, the enzyme turned out to be sensitive towards detergents (0.5% *w*/*v*, Triton-X100 and Tween-40).

Additionally, the enzyme was incubated with resorcinol (10 mM), phloroglucinol (10 mM), DAPG (15 mM), and MAPG (15 mM) prior to biotransformation. In case of resorcinol and phloroglucinol, a slight increase in residual activity was observed (105 and 107% respectively, while for MAPG and DAPG, a slight decrease was noticed, 92–93% (Fig. 9).

Enzyme activity was also investigated in the presence of the chloride salts of Ca^2+^, Mg^2+^, Zn^2+^, Cu^2+^, Co^2+^, Mn^2+^, Sr^2+^, or Ni^2+^ at 5 or 8 mM concentration and compared to a control reaction in the absence of the metal (Fig. S9). To avoid precipitation of the metal with a buffer component (e.g., phosphate), all reactions were performed in HEPES-buffer (50 mM, pH 7.5) using solutions and ATase containing cell-free extract prepared in the same buffer. Most of the bivalent metals were tolerated by the ATase without having noteworthy effects on the enzyme. Only Cu^2+^ led to significantly reduced conversions.

## Discussion

Three *Pseudomonas* strains, *P. protegens* DSM19095, *P. brassicacearum* DSM13227, and *P. fluorescens* Pf-5 ATCC BAA-477 with acyltransferase activity were identified to catalyze the reversible disproportionation of two molecules of natural substrate MAPG into PG and DAPG. In contrast to other acyltransferases, the ATase involved in the biosynthesis of DAPG is independent of the coenzyme A (CoA) cofactor (Hayashi et al. 2012). No product was detected in the absence of the cell preparations, indicating that product formation can be assigned to the acyltransferase activity present in the mentioned *Pseudomonas* strains. As *P. fluorescens* Pf-5 was the least active strain under the conditions employed, subsequent investigations of the substrate spectrum were exclusively performed with *P. brassicacearum* and *P. protegens*. Although higher activities were obtained with the recombinant enzyme from *P. protegens* (*Pp*ATaseWT) compared to *P. brassicacearum* (*Pb*ATaseWT), optimum expression conditions were established for both enzyme preparations.

In order to obtain soluble and active protein, parameters such as temperature, culture media, and time were investigated. Major differences in terms of functional expression of both ATases were noticed. Equally important was the type of the expression medium. LB-media turned out to be the best compromise between cell densities and protein activity. A time period of 21 h was chosen as an optimum for the expression of the *Pp*ATaseWT and 6–7 h for the *Pb*ATaseWT in LB-media.

For expression of the heterotrimeric enzyme in *E. coli*, it was shown that the introduction of ribosomal binding site upstream of the *phl* coding sequences as well as the codon-optimization significantly improved the expression of the ATase in *E. coli*. Furthermore, it is worth mentioning that almost no inclusion bodies were formed under these conditions indicating that the proteins *PhlA*, *PhlC*, and *PhlB* were expressed in a soluble form. Despite the high-level expression observed for T7-promoter, formation of inclusion bodies was observed at any investigated temperature. From these observations, it can be deduced that a weaker promoter, such as the *tet*-promoter in pEG332 is much better suited for the overexpression of the ATase than the strong T7-system in pCAS1.

During optimization, it was noticed that the size and shape of the shaking flasks did not significantly influence the ATase expression, although significant variation between experiments were observed (Table 1, entry 3, 4). Optimum expression conditions can be summarized as follows: *E. coli* BL21 worked best in LB-media, non-baffled shake flasks (5 L) with 200 μg L^−1^ AHTC at 30 °C and 140 rpm. Optimization of the expression conditions, as well as codon-optimization, let to significantly improved enzyme activities compared to *Pp*ATaseWT (Fig. 4a, black vs. gray circles).

The type of cell/enzyme treatment had also a significant impact on the activity of the catalyst preparation, while the storage temperature seemed to play a minor role. Inert storage did not prevent from activity loss, which leads to the assumption that oxygen is not involved in the observed degradation. Both enzyme preparations, *Pp*ATaseWT, and *Pb*ATaseWT showed similar behavior upon long-term storage. Finally, after proving the negative effect of lyophilization, the enzymes were subsequently stored at − 20 °C as a cell-free extract or as PBS-treated lyophilized cells.

It is worth mentioning, that the enzyme is not only sensitive to lyophilization, but also to increased temperatures. From thermostability studies, it can be concluded, that ATase is temperature-sensitive, and 35 °C seems to be the best compromise between stability and activity.

The *Pp*ATaseCH showed the highest activity at slightly alkaline pH, as product formation was most efficient when reactions were carried out between pH 7.5 and 8.5 KPi-buffer (Fig. 4b). Alkaline pH resulted in lower enzyme activity; however, it has to be mentioned that CHES-buffer was obviously less suitable for the enzyme because overall conversions were considerably lower compared to reactions performed in KPi-buffer. Taking into account that pH 8.0 constitutes the upper operational limit of the KPi buffer salt, pH 7.5 was used as best pH for the substrates investigated here.

High co-solvent tolerance is particularly useful if the solubility and thus the availability of hydrophobic substrates in the reaction medium has to be increased. Furthermore, product separation and work-up procedures can be significantly facilitated by integrating a suitable co-solvent into the reaction system. After mapping the general co-solvent tolerance of the ATase, potential solubility issues may be addressed by implementing one of the tolerated solvents, preferably MTBE, diethyl ether, or EtOAc.

Small chemical compounds may affect the enzymatic activity, either by specifically interacting with distinct amino acids such as active-site residues or by influencing the overall structure. Some of tested compounds react with SH-groups (DTT, β-Met, IAA, *p*CMB), OH-moieties (PMSF) or NH-groups (DEPC). After treatment with *p*CMP or IAA, which are known for their ability to covalently bind to thiol moieties, the enzyme activity was abolished. This may support the assumption that cysteine (most likely cysteine 88) is involved in the acyl transfer. Furthermore, the enzyme turned out to be sensitive towards detergents (0.5% *w*/*v*, Triton-X100, and Tween-40). Detergents tend to alter the quaternary and tertiary structure of proteins, and may even lead to denaturation. At this point, it has to be mentioned that none of the investigated reagents are ATase-specific inhibitors; thus, the influence on other structural elements besides active-site residues might be considered as well. Nevertheless, this study allows a rough estimation of possible hot-spots, responsible for the observed catalytic action of the ATase.

Although phenols are known for their denaturating properties, resorcinol or phloroglucinol did not cause any loss of activity but actually improved activity, while MAPG and DAPG decreased activity only slightly (92–93%, Fig. 9); thus, apparently these reagents had no significant denaturing effect under the conditions investigated.

The effects of bivalent metals on the stability and activity of proteins is well studied and generally depends on the ionic radii of the metal and the coordination geometries it can adopt (Yang et al. 2003; Coolbear et al. 1992). Neutral impact of investigated bivalent alkaline-earth and transition metals on the biotransformation confirmed, that ATase catalyzes C-acyl transfer reactions independently of a metal cofactor. Only Cu^2+^ led to significantly reduced conversions, indicating enzyme inhibition.

In summary, identification of suitable expression conditions using *E. coli* BL21 cells, optimum temperature (30 °C for *Pp*ATaseWT and 25 °C for *Pb*ATaseWT), LB-medium, expression time (21 h for *Pp*ATaseWT and 7 h for *Pb*ATaseWT), and cultivation in 5-L non-baffled flask led to catalyst preparations of highest activity. We identified KPi-buffer pH 7.5 as the most suitable reaction medium, while the addition of co-solvents and bivalent metals had no notable influence on the performance of the ATase. Introduction of ribosomal binding sites upstream of the *phl* genes and the codon-optimization significantly improved the expression of the ATase in *E. coli* compared to the wild-type genes.

## Electronic supplementary material

ESM 1 (PDF 1431 kb)
