# Supplementary material for: Molecular cloning, expression, and characterization of acyltransferase from Pseudomonas protegens
Source: Appl Microbiol Biotechnol. 2018 May 12;102(14):6057–68. doi: 10.1007/s00253-018-9052-z (PMC6013524; doi:10.1007/s00253-018-9052-z)
Supplement: Supplementary file 1 — (PDF 1431 kb) [file 253_2018_9052_MOESM1_ESM.pdf]

Applied Microbiology and Biotechnology

Supplementary materials

Molecular cloning, expression and characterization of acyltransferase from *Pseudomonas protegens*

Nina G. Schmidt,[1,2] Anna Żądło-Dobrowolska,[2] Valerie Ruppert,[2] Christian Höflehner,[2] Birgit Wiltschi,[1] Wolfgang Kroutil*[1,2]

[1]ACIB GmbH, Graz, Austria

[2]Institute of Chemistry, University of Graz, NAWI Graz, BioTechMed Graz, Graz, Austria

*Corresponding Author: wolfgang.kroutil@uni-graz.at;

Tel.: +43/(0)316/380-5350; Fax.: +43/(0)316/380-9840

Supporting Information

Table of Contents

1. Screening of *Pseudomonas* wildtype strains 3
2. Plasmid construction of recombinant ATases 4
2.1. Protein sequence-alignment of PhlA, PhlC and PhlB 5
2.2. Cloning of *Pb*ATaseWT (pEG330) and *Pp*ATaseWT (pEG331) 8
2.3. Cloning of *Pp*ATaseCH (pEG332) 9
2.4. Cloning of *Pp*ATaseCH (pCAS1) 14
3. SDS-PAGE analysis 15
4. Alternative ATase Preparations and Storage Types 15
5. Modified procedure to test the impact of bivalent metals 16
6. Modified procedure to test the impact of inhibitors/additives 18
7. Synthesis of 2,4-diacetylphloroglucinol (DAPG, 5) 18
References 18

1. Screening of *Pseudomonas* wildtype strains

32 strains from the in-house culture collection (Table S1) were tested for their ability to catalyze a reversible acetylation and deacetylation of MAPG (Figure S1).

Table S1. *Pseudomonas* strains investigated.

Entry	Strain	Designation
1	*Pseudomonas acidovorans*	ATCC 17438
2	*Pseudomonas aureofaciens*	ATCC 43051
3	*Pseudomonas brassicacearum*	DSM 13227
4	*Pseudomonas chlororaphis*	ATCC 9447
5	*Pseudomonas cichorii*	DSM 50259
6	*Pseudomonas dehalogenans R*	FCC 162
7	*Pseudomonas elodea*	ATCC 31461
8	*Pseudomonas fluorescens*	DSM 50106
9	*Pseudomonas fluorescens*	ATCC 17571
10	*Pseudomonas fluorescens*	ATCC 49838
11	*Pseudomonas fluorescens*	NRRL B 00010
12	*Pseudomonas fluorescens* Pf-5	ATCC BAA-477
13	*Pseudomonas fragi*	DSM 3456
14	*Pseudomonas marginalis*	FCC 177
15	*Pseudomonas mephitica*	FCC 178
16	*Pseudomonas oleovorans*	ATCC 29347
17	*Pseudomonas ovalis*	ATCC 00950
18	*Pseudomonas pavonacea*	NRRL B 00969
19	*Pseudomonas protegens*	DSM 19095
20	*Pseudomonas pseudoalcaligenes*	DSM 10086
21	*Pseudomonas putida*	FCC 145
22	*Pseudomonas putida*	ATCC 17453
23	*Pseudomonas putida*	ATCC 47054
24	*Pseudomonas putida*	DSM 12264
25	*Pseudomonas rhodesiae*	FCC 179
26	*Pseudomonas* sp.	DSM 6978
27	*Pseudomonas* sp.	DSM 12877
28	*Pseudomonas* sp.	NCIMB 11753
29	*Pseudomonas stutzeri*	DSM 17083
30	*Pseudomonas syringae*	DSM 50272
31	*Pseudomonas syringae*	DSM 1241
32	*Pseudomonas thermotolerans*	DSM 14292

Conditions: Lyophilized cells of the respective *Pseudomonas* strain (20 mg), KPi-buffer (50 mM, pH 7.5), MAPG (50 mM, forward reaction) or alternatively PG and DAPG (50 mM, reverse reaction), 3 h, 30 °C and 500 rpm.

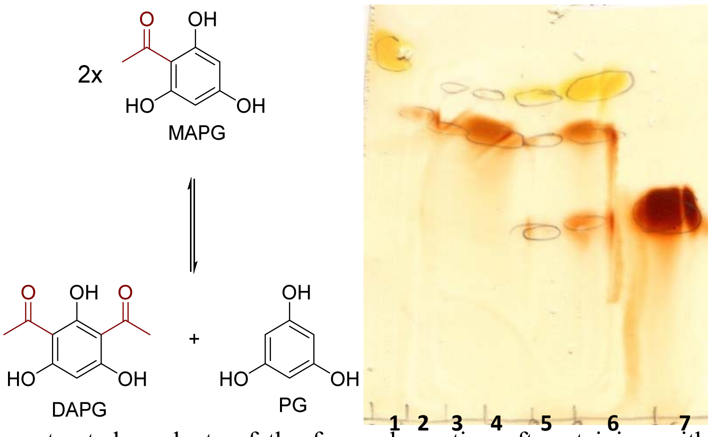

Figure S1. TLC of the extracted products of the forward reaction after staining with cinnamaldehyde*HCl. References: DAPG (**5**, lane 1), MAPG (**6**, lane 2), PG (**7**, lane 7). Reactions (50 mM, **6**): *P. protegens* DSM19095 (lane 3-4); *P. brassicacearum* DSM13227 (lane 5-6).

2. Plasmid construction of recombinant ATases

Primer sequences and plasmids used in this study are listed in Table S2. To construct the expression constructs *Pp*ATaseWT and *Pb*ATaseWT, the genomic DNA of the respective *Pseudomonas* wild-type served as template to amplify the ATase-encoding operon *phlACB* by PCR. The PCR products were digested (*Kpn*I/*Bam*HI), purified and ligated into target vector pASK-Iba3plus. The obtained expression vectors carry the ATase encoding genes *phlACB* under the control of the P_{Tet} promoter. To construct the recombinant *Pp*ATaseCH with the optimized sequence, the ATase encoding open-reading frames *phlA*, *phlC* and *phlB* of *P. protegens* were codon-optimized by manually matching the codon-frequency of the *Pseudomonas* wild-type with *E. coli*. To achieve this goal, codon-usage tables for *Escherichia coli* B and *Pseudomonas fluorescens* were obtained from the Kazusa-database (http://www.kazusa.or.jp/codon/). Ribosomal binding sites suitable for *E. coli* were introduced upstream of each start codon of each *phl* gene. The optimized *phl* genes were purchased as gene fragments (gBlocks©) and assembled with the double-digested pASKIBA3plus backbone (*Eco*RI/*Hind*III) by overlap extension-PCR (OE-PCR) and subsequent Gibson cloning (Gibson Assembly® master mix). The final expression vector carried the *E. coli* codon-optimized ATase encoding genes *phlACB* under the control of the P_{Tet} promoter.

Table S2. Plasmids and primers employed in this study. Mutagenized codons are shown in bold, restriction site are underlined. Ribosomal binding sites are shown in lowercase letters.

**Plasmids**	**Origin (GenBankID)**	**Description/Comments**
pASKIBA3plus	IBA-Lifescience	P_{Tet}, Amp^r, $ColE1_{ori}$, C-terminal StrepTag
pEG331	this study (CP003190.1)	Wild-type-derived *phlACB* genes of *P. protegens* DSM19095, isolated from genomic DNA by PCR; PCR primers: *Pp*WT-Fow/Rev.
pEG330	this study (KY173354)	Wild-type-derived *phlACB* genes of *P. brassicacearum* DSM13227, isolated from genomic DNA by PCR; PCR primers: *Pb*WT-Fow/Rev
pEG332	this study (KY173355)	Codon-optimized gene fragments *phlA, phlC* and *phlB* based on *phlACB* from *P. protegens* DSM19095, assembled by Gibson cloning and overlap-extension PCR. PCR primers: OE1-4ATaseCH-Fow/Rev.
**Primers**	**Origin**	**Sequence (5'→3')**
*Pp*WT-Fow	Eurofins	ATATAGGTACCATGAACGTGAAAAAGATAGGTATTG
*Pp*WT-Rev	Eurofins	ATATAGGATCCTTATATATCGAGTACGAACTTATAAG
*Pb*WT-Fow	Eurofins	ATATAGGTACCATGAATAAAGTAGGAATTGTG
*Pb*WT-Rev	Eurofins	ATATAGGATCCTTATTTCACCAGTACAAACTTATAG
OE1ATaseCH-Fow	IDT	ATATAAGAATTCaaggagatatacataTGATGAATGTGAAGAAAATAGGTATCGTTAGC
OE2ATaseCH-Fow	IDT	CGCTGACCGCGTACCTCTAAGGTACCaaggagatatacataTGATGTGCGCACGTCGCG
OE3ATaseCH -Rev	IDT	TGCGCACATCAtatgtatatctccttGGTACCTTAGAGGTACGCGGTCAGCGCATAATC
OE4ATaseCH -Rev	IDT	ATATATGAATTCGCCGAGACGGCCATG
**Bacterial Strains**	**Origin (Strain ID)**	**GenBankID (*phlACB* gene locus)/comments**
Pseudomonas brassicacearum	DSMZ (DSM13227)	LT629713.1 (bp: 1051432-1054193) / *phlACB* from *P. brassicacearum* BS3663 are 100% to *phlACB* from *P. brassicacearum* DSM13227
Pseudomonas protegens	DSMZ (DSM19095)	CP003190.1 (bp: 6560049-6562816) / other designation: CHA0
Pseudomonas fluorescens Pf5	ATCC (ATCC BAA-477)	CP000076.1 (bp: 6766435-6769202)

2.1. Protein sequence-alignment of PhlA, PhlC and PhlB

The *phlACB* operon from *P. protegens* DSM19095 and *P. brassicacearum* DSM13227 was amplified from the genomic DNA using primer sequences which were identified in a BLAST-search (Table S3).

Table S3. BLAST-search results.

**Entry**	**Organism**	**Description**	**GenBank accession no.**	**Seq. identity [%]**
1	*Pseudomonas* sp. YGJ3	*phlACBDEFGHI* complete cds	AB636682.1	100
2	*P. protegens* CHA0	complete genome	CP003190.1	99
3	*P. brassicacearum* NFM241	complete genome	CP002585.1	80
4	*P. fluorescens* J2	*phlA, phlB, phlC, phlD* complete cds	JN561597.1	80

Multiple sequence alignments of the *Phl*-subunits were performed using the T-COFFEE multiple sequence alignment program provided by EMBL-EBI.[1] *PhlA*, *PhlC* and *PhlB* originating from *P. protegens* (*Pp*), *P. brassicacearum* (*Pb*) and *P. fluorescens* Pf-5 (*Pf*5) were aligned to the *Phl*-subunits of *Pseudomonas* sp. YGJ3. The rendering of the sequence alignments was performed with ESPript 3.0 (http://espript.ibcp.fr).[2] The secondary structure elements of individual *Phl*-subunits from *P. protegens,* as present in the crystal structure, are depicted above the alignments (Figure S2-S4).

```
               1        10        20        30        40        50
YGJ3-PhlA    MNVKKIGIVSYGAGIPVCRLKVQEVINVWKNTDLKLVEENLGVTERAVLQ
Pp-PhlA      MNVKKIGIVSYGAGIPVCRLKVQEVINVWKNTDLKLVEENLGVTERAVLQ
Pb-PhlA      MN..KVGIVSYGAGIPVCRLKVDDVIQVWKNTDLSLVKGQLGVLERAVLQ
Pf5-PhlA     MNVKKIGIVSYGAGIPVCRLKVQEVINVWKNTDLKLVEENLGVTERAVLQ

                      60        70        80        90       100
YGJ3-PhlA    PDEDVITLGVLAAQRALDKVPGHQIEALYLGTCTNPYDSRASASIILEML
Pp-PhlA      PDEDVITLGVLAAQRALDKVPGHQIEALYLGTCTNPYDSRASASIILEML
Pb-PhlA      PDEDVITLGVLAAQRALDKAPPCSLEALYLGTCTNPYDSRASAAIILEML
Pf5-PhlA     PDEDVITLGVLAAQRALDKVPGHQIEALYLGTCTNPYDSRASASIILEML

                     110       120       130       140       150
YGJ3-PhlA    GSGYDAYCADVQFAGKSGTSALQICQALVASGMTGSALAIGADTINRNTA
Pp-PhlA      GSGYDAYCADVQFAGKSGTSALQICQALVASGMTGSALAIGADTINRNTA
Pb-PhlA      GCGYDAFCADVQFAGKSGTSALQIAYALVASGMVGNALAVGADTINRNTA
Pf5-PhlA     GSGYDAYCADVQFAGKSGTSALQICQALVASGMTGSALAIGADTINRNTA

                     160       170       180       190       200
YGJ3-PhlA    PGDLTESYAGAGAAALLIGSQDVIAEFDASFSCAADVADNIRPQGDRYIR
Pp-PhlA      PGDLTESYAGAGAAALLIGSQDVIAEFDASFSCAADVADNIRPQGDRYIR
Pb-PhlA      PGDLTESYAGAGAAALLLGTENVIAHFDASFSCAADVADNIRPQGDRYIR
Pf5-PhlA     PGDLTESYAGAGAAALLIGSQDVIAEFDASFSCAADVADNIRPQGDRYIR

                     210       220       230       240       250
YGJ3-PhlA    SGMGLGSDKNSIGLEDQTRRAAEGLMAKLHTSPADYDYVVFQQNLVSTPY
Pp-PhlA      SGMGLGSDKNSIGLEDQTRRAAEGLMAKLHTSPADYDYVVFQQNLVSTPY
Pb-PhlA      SGMGLGSDKNSIGLEDQTRRAASGLMAKIHAQAGDFDYVVFQQNLVSTPY
Pf5-PhlA     SGMGLGSDKNSIGLEDQTRRAAEGLMAKLHTSPADYDYVVFQQNLVSTPY

                     260       270       280       290       300
YGJ3-PhlA    SLAKHLGFNPKQVEPGIYAGNVGDAGSASPLLGLINVLDQARPGQKILLV
Pp-PhlA      SLAKHLGFNPKQVEPGIYAGNVGDAGSASPLLGLINVLDQARPGQKILLV
Pb-PhlA      SLGKHLGFTTAQIEPGIYAQSVGDAGAASPLLGLVNVLDQARPGERILVV
Pf5-PhlA     SLAKHLGFNPKQVEPGIYAGNVGDAGSASPLLGLINVLDQARPGQKILLV

                     310       320       330       340       350
YGJ3-PhlA    SYGFGAGSDAIALTVTDAIEQYQKHNKPLRELLESKIYVDYGTSIKYEFK
Pp-PhlA      SYGFGAGSDAIALTVTDAIEQYQKHNKPLRELLESKIYVDYGTSIKYEFK
Pb-PhlA      SYGFGAGSDAIALTVTDAIEAYQKTNVPLRTLLEDKYYVDYGTSIKYEFK
Pf5-PhlA     SYGFGAGSDAIALTVTDAIEQYQKHNKPLRELLESKIYVDYGTSIKYEFK

                     360
YGJ3-PhlA    YLRADYALTAYL
Pp-PhlA      YLRADYALTAYL
Pb-PhlA      YLRPDYALTAYL
Pf5-PhlA     YLRADYALTAYL
```

Figure S2. Protein sequence alignment of PhlA. Sequences of PhlA from *P. protegens* (*Pp*), *P. brassicacearum* (*Pb*) and *P. fluorescens* (*Pf*-5) were aligned to *Pseudomonas* sp. YGJ3.

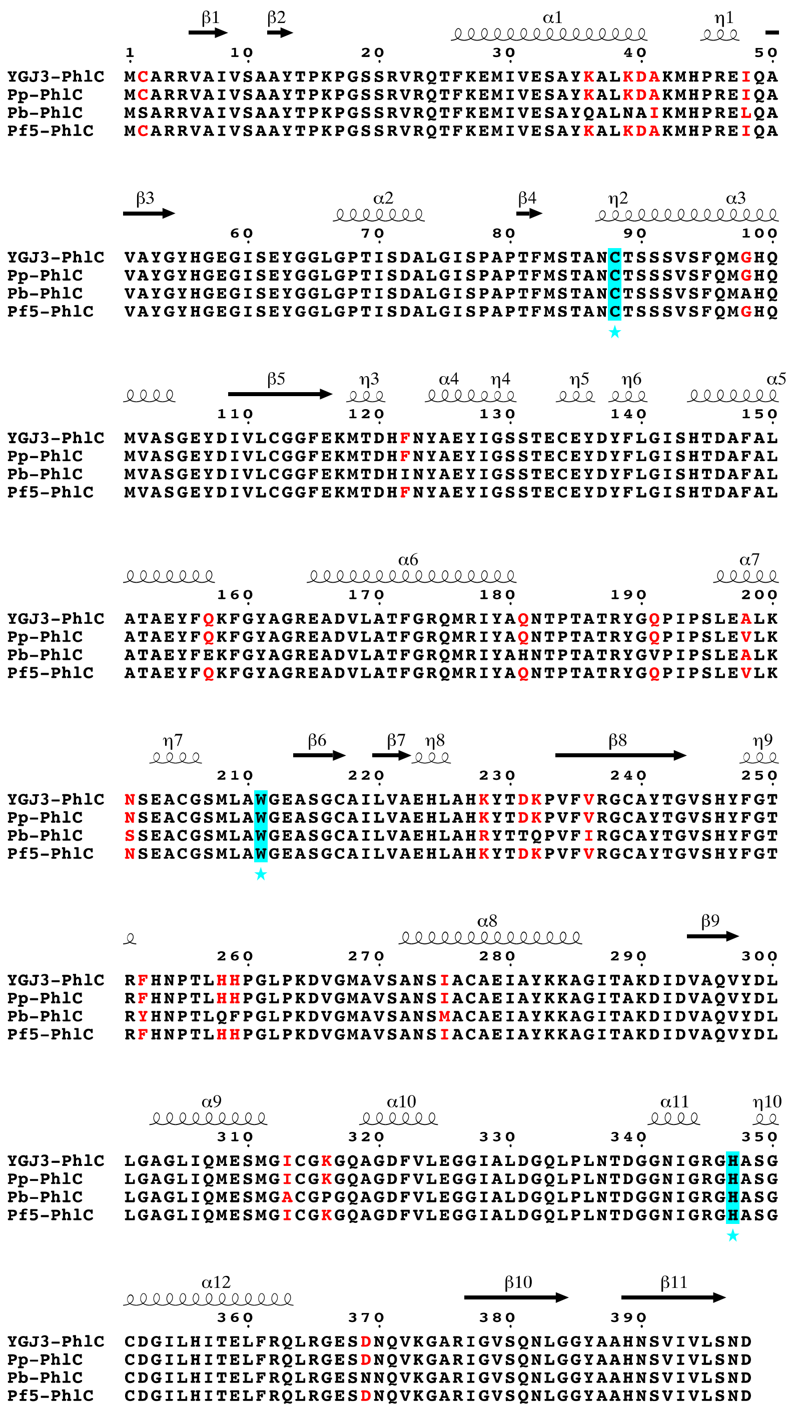

Figure S3. Protein sequence alignment of *PhlC*. Sequences of *PhlC* from *P. protegens* (*Pp*), *P. brassicacearum* (*Pb*) and *P. fluorescens* (*Pf*-5) were aligned to *Pseudomonas* sp. YGJ3.

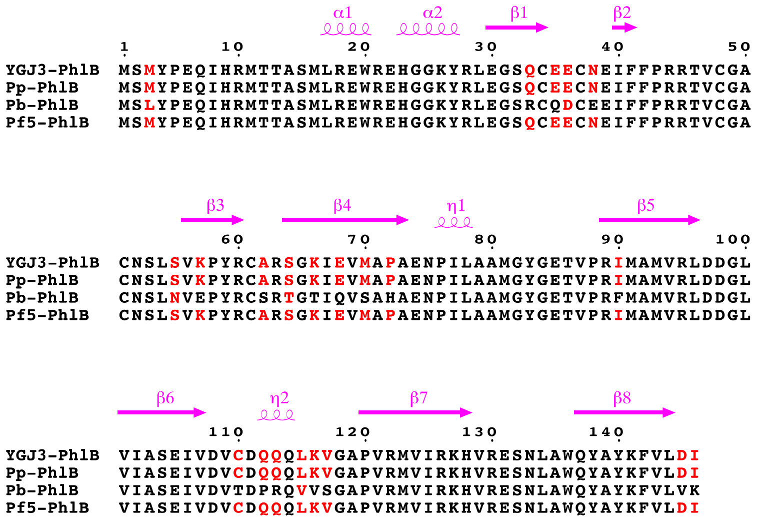

Figure S4. Protein sequence alignment of *PhlB*. Sequences of *PhlB* from *P. protegens* (*Pp*), *P. brassicacearum* (*Pb*) and *P. fluorescens* (*Pf*-5) were aligned to *Pseudomonas* sp. YGJ3.

2.2. Cloning of *Pb*ATaseWT (pEG330) and *Pp*ATaseWT (pEG331)

PCR-amplification of the wild-type *phlACB* operon. The ATase encoding *phlACB* operon (approx. 2770 bp) was amplified from the genomic DNA of *P. protegens* or *P. brassicacearum*. The genomic DNA was isolated according to the manufacture's protocol (PureLink® Genomic DNA Minikit, Thermo Fischer). The following primers were used (restriction site underlined):

*Pb*ATase-FW: 5'-ATATA<u>GGTACC</u>ATGAATAAAGTAGGAATTGTG-3'

*Pb*ATase-REV: 5'-ATATA<u>GGATCC</u>TTATTTCACCAGTACAAACTTATAG-3'

*Pp*ATase-FW 5'-ATATA<u>GGTACC</u>ATGAACGTGAAAAAGATAGGTATTG-3'

*Pp*ATase-REV 5'-ATATA<u>GGATCC</u>TTATATATCGAGTACGAACTTATAAG-3'

The PCR reaction mixture consisted of the following components:

26 µL H_2O sterile

10 µL Phusion GC buffer (5×)

1 µL template (genomic DNA)

5 µL primer forward (5 nmol µL^{-1})

5 µL primer reverse (5 nmol µL^{-1})

1.5 µL DMSO (3 vol%)

1 µL dNTPs (0.2 nmol µL^{-1})

0.5 µL Phusion DNA polymerase (2 U µL^{-1})

The following PCR program was used:

1× 98 °C 2:00 min

25× {	98 °C	0:20 min
	58 °C	0:15 min
	72 °C	2:00 min
1×	72 °C	3:00 min
	4 °C	∞

Column purification of the PCR-products was performed (Qiagen®-PCR purification kit). The approximate concentration of the PCR products was determined by agarose gel electrophoresis.

Restriction and ligation. The PCR products (0.2 µg) and the pASK-IBA3plus vector backbone (1 µg) were digested with *Kpn*I and *Bam*HI (FastDigest, Thermo Fischer). The DNA was gel-purified prior to ligation.

The ligation consisted of the following components:

12 µL H_2O sterile
4 µL insert
1 µL vector
2 µL ligation buffer (10×)
1 µL T4 ligase

The reaction was incubated overnight at 4 °C. 5 µL of the ligation mix was transformed into *E. coli* DH5α and streaked onto LB plates containing 100 µg mL^{-1} ampicilin for selection. Isolated plasmids (QIAprep Spin Miniprep Kit, Qiagen®) of randomly picked clones were controlled by restriction digest and sequencing.

2.3. Cloning of *Pp*ATaseCH (pEG332)

The genes *phlA*, *phlC* and *phlB* originating from *P. protegens* were codon-harmonized by manually matching the codon-frequency of *Pseudomonas* to *E. coli*. Ribosomal binding sites (RBS) were introduced at the 5'-end of each *phl* gene. The optimized *phl** genes were ordered as gene strings (gBlocks®, IDT). Cloning of the gene strings into the pASK-IBA3plus expression vector was accomplished by Gibson assembly and overlap extension PCR (OExPCR).

Gibson cloning. A four-fragment Gibson assembly [5] between the pASK-IBA3plus vector (*Eco*RI/*Bam*HI digested, gel-purified) and the gene strings, *phlA**, *phlC** and *phlB** was carried out (Figure S5 and Table S4) using the Gibson assembly master mix® (New England Biolabs).

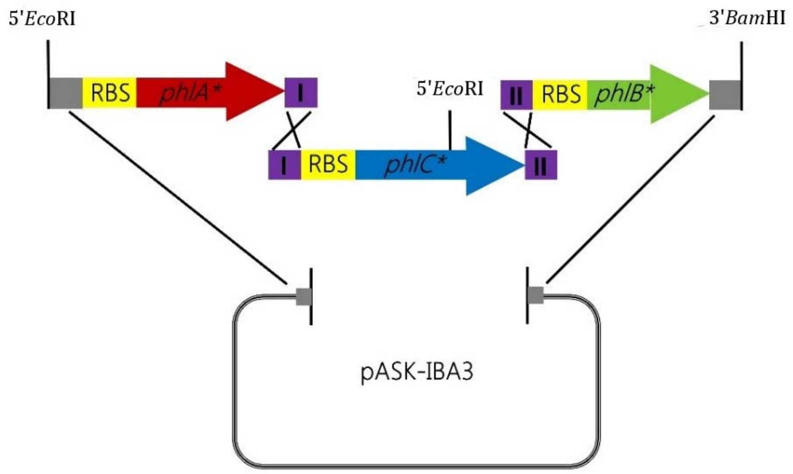

Figure S5. Cloning strategy. Four-fragment Gibson assembly of the optimized *phl** genes and the pASK-IBA3 backbone.

The following amounts were applied (Table S4):

Table S4. Calculations for the Gibson assembly sample preparation.

Fragment	Size (bp)	pmol	Mass (ng)
pASK-IBA3plus vector	3226	0.048	100
*phlA**	1238	0.048	38.4
*phlC**	1346	0.048	41.7
*phlB**	590	0.048	18.3

The assembly was performed according to the manufacture's protocol followed by direct transformation into *E. coli* DH5α.

Colony-PCR. Gibson assembled clones were verified by colony-PCR using primers which flank the desired insert:

IBA3-FW: 5'-GAGTTATTTACCACTCCCT-3'
IBA3-REV: 5'-CGCAGTAGCGGTAAACG-3'

The PCR reaction mixture consisted of the following components:

7.5 µL H$_2$O sterile
12.5 µL DreamTaq PCR-mastermix (2×)
2.5 µL primer forward (5 nmol µL^{-1})
2.5 µL primer reverse (5 nmol µL^{-1})

The following PCR program was used:

1×	95 °C	3:00 min
25×	94 °C	0:30 min
	58 °C	0:30 min
	72 °C	2:50 min
1×	72 °C	5:00 min
	4 °C	∞

Selected clones were restreaked onto LB plates containing 100 µg mL^{-1} ampicillin for selection and the isolated plasmids were sequenced. Misassembled DNA stretches within *phlA** and *phlC** of the Gibson assembled plasmid pEG332_C20 (Figure S6, a) were corrected by OEx PCR to establish the correct *phlACB** construct (pEG332).

OExPCR. The OExPCR consisted of 3 steps: (i) extension PCR 1 & 2, (ii) overlap extension PCR, (iii) purification PCR [4] (Figure S6, b).

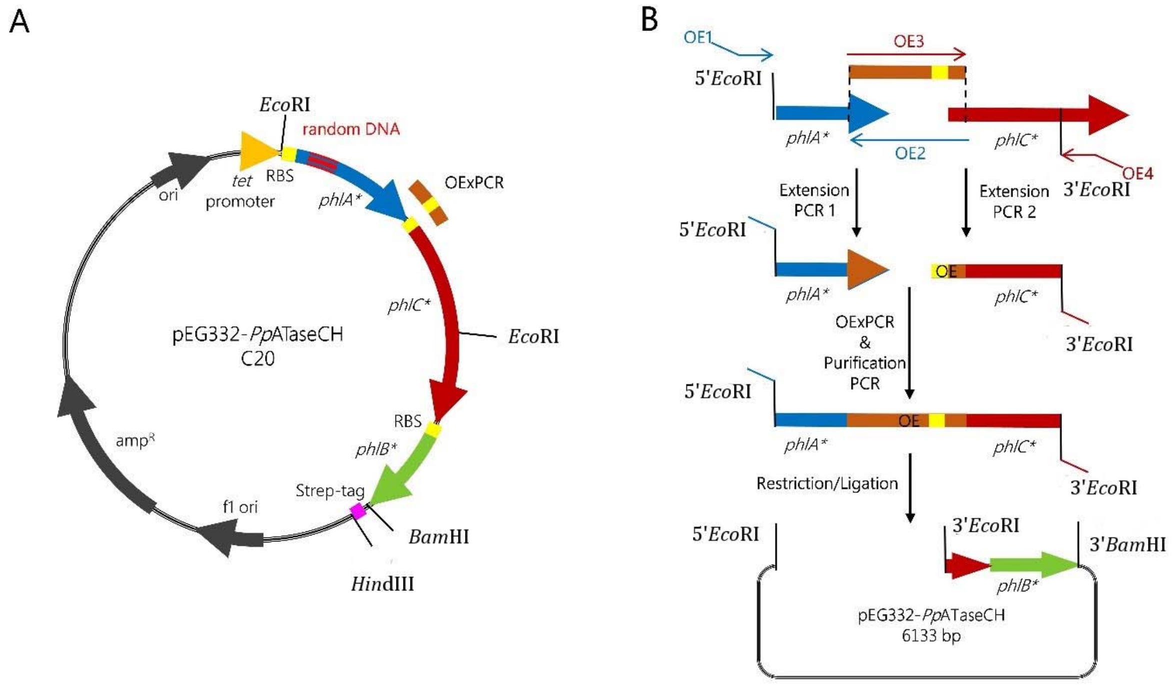

Figure S6. Cloning strategy to "repair" the defective pEG332_C20 plasmid. (**a**) Defective pEG332_C20 obtained *via* Gibson assembly containing random DNA stretches (≈100 bp) within *phlA**. (**b**) Overview of the OExPCR to establish the correct *phlACB** construct (pEG332).

(i) Extension PCR. Areas of homology (OE-sequences) required for the subsequent overlap PCR were introduced to the flanking regions of the *phlA** and *phlC** gene strings. The following primers were used (restriction sites underlined; RBS small letters):

OE1ATaseCH-FW:
5'-ATATAAGAATTCaaggagatatacataTGATGAATGTGAAGAAAATAGGTATCGTTAGC-3'
OE2ATaseCH-REV:
5'-CGCTGACCGCGTACCTCTAAGGTACCaaggagatatacataTGATGTGCGCACGTCGCG-3'
OE3ATaseCH-FW:
5'-TGCGCACATCAtatgtatatctccttGGTACCTTAGAGGTACGCGGTCAGCGCATAATC-3'
OE4ATaseCH-REV:
5'-ATATATGAATTCGCCGAGACGGCCATG-3'

The PCR reaction mixture consisted of the following components:

25 µL H$_2$O sterile
10 µL Phusion GC buffer (5×)
2.5 µL template (2.5 ng, *phlA** or *phlC** gene strings)
2.5 µL primer forward (5 nmol µL^{-1})
2.5 µL primer reverse (5 nmol µL^{-1})
1.5 µL DMSO (3 vol%)
5 µL dNTPs (0.2 nmol µL^{-1})
0.5 µL Phusion DNA polymerase (2 U µL^{-1})

The following PCR program was used:

1×	95 °C	0:45 min
25×	94 °C	0:10 min
	60 °C	0:20 min
	72 °C	0:40 min
1×	72 °C	3:00 min
	4 °C	∞

The products of the extension PCR (*phlA*_OE & phlC*_OE*) were gel-purified, blunt-end ligated into pJET1.2 according to the manufacture's protocol (CloneJET-PCR Cloning Kit, Thermo Scientific) and transformed into *E. coli* DH5α. Isolated plasmids of selected clones were sent for sequencing.

(ii) Overlap PCR. The products of the extension PCR, *phlA*_OE* and *phlC*_OE*, were spliced together in a primerless overlap PCR yielding *phlAC*_OE* (15 cycles). The amounts of template DNA were calculated based on 10.0 ng of the biggest fragment using the following equation:

$$m_{Insert\_small} = \frac{m_{insert\_big}[ng] * size_{insert\_small}[bp]}{size_{insert\_big}[bp]}$$

The PCR reaction mixture consisted of the following components:

32.3 µL H$_2$O sterile
10 µL Phusion GC buffer (5×)
1 µL template 1 (10 ng, *phlA*_OE*)
0.75 µL template 2 (7.5 ng, *phlC*_OE)*
1.5 µL DMSO (3 vol%)
4 µL dNTPs (0.2 nmol µL^{-1})
0.5 µL Phusion DNA polymerase (2 U µL^{-1})

The following PCR program was used:

1×	98 °C	0:30 min
15×	98 °C	0:10 min
	60 °C	0:20 min
	72 °C	0:40 min
1×	72 °C	7:00 min
	4 °C	∞

(iii) Purification PCR. Flanking primers (OE1ATaseCH-FW & OE4ATaseCH, *vide supra*) were directly added to the overlap PCR mix in order to amplify the spliced gene product *phlAC_OE* (20 cycles).

The PCR reaction mixture consisted of the following components:

50 µL overlap-PCR mix
5.65 µL H_2O sterile
4 µL Phusion GC buffer (5×)
4 µL primer forward (5 nmol μL^{-1})
4 µL primer reverse (5 nmol μL^{-1})
0.6 µL DMSO (3 vol%)
1.25 µL dNTPs (0.2 nmol μL^{-1})
0.5 µL Phusion DNA polymerase (2 U μL^{-1})

The following PCR program was used:

1×	98 °C	0:30 min
20×	98 °C	0:10 min
	60 °C	0:20 min
	72 °C	0:40 min
1×	72 °C	7:00 min
	4 °C	∞

The entire reaction mixture was loaded onto an agarose gel and the desired gene product *phlAC*_OE* (approx. 1986 bp) was gel-purified (Figure S7).

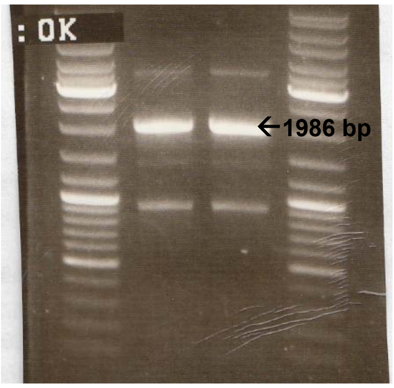

Figure S7. Different products obtained by OExPCR. The strong band (≈1986 bp) belongs to the desired gene product *phlAC*_OE*.

Restriction and ligation. The misassembled DNA stretch in pEG332_C20 was removed by restriction digest (*Eco*RI). The remaining backbone pEG332_C20 was recovered and gel-purified. Ligation with *phlAC*_OE* finally established the correct *phlACB** construct (pEG332).

The ligation consisted of the following components:

8.7 µL	insert (186 ng, *phlAC*_OE*)
4 µL	vector (100 ng, pEG332_C20)
4.3 µL	H_2O sterile
2 µL	ligation buffer (10×)
1 µL	T4 ligase

The reaction was incubated overnight at 4 °C. 5 µL of the ligation mix was transformed into *E. coli* DH5α and streaked onto LB plates containing 100 µg mL^{-1} ampicillin for selection. Isolated plasmids of randomly picked clones were controlled by restriction digest and sequencing.

2.4. Cloning of *Pp*ATaseCH (pCAS1)

Restriction and ligation. Cloning of the codon-harmonized *phlACB** construct from pEG332 into the T7-regulated pCAS1 expression vector was accomplished by restriction digest with *Eco*RI and *Bam*HI (1 µg DNA). The fragments were gel-purified prior to ligation.

The triple ligation (1:1:1 ratio) consisted of the following components:

6.1 µL	insert 1 (30.3 ng)
4.6 µL	insert 2 (60.6 ng)
2.3 µL	vector (100 ng)
4 µL	H_2O sterile
2 µL	ligation buffer (10×)
1 µL	T4 ligase

The reaction was incubated overnight at 4 °C. 5 µL of the ligation mix was transformed into *E. coli* DH5α and streaked onto LB plates containing 100 µg mL^{-1} ampicillin for selection. Isolated plasmids of randomly picked clones were controlled by restriction digest and sequencing.

3. SDS-PAGE analysis

Cell-free extract was analyzed by SDS-PAGE (Figure S8)

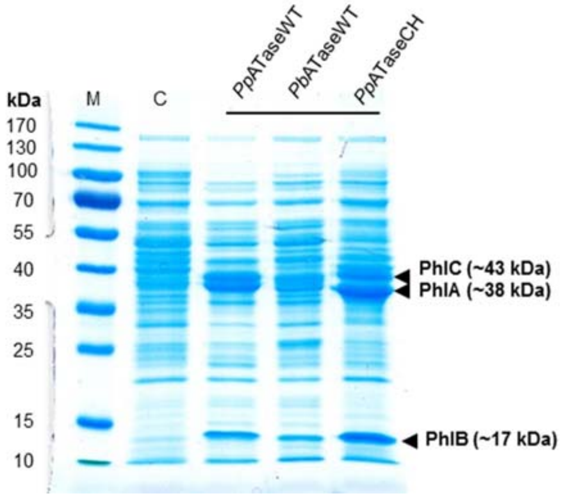

Figure S8. SDS-PAGE analysis of the cell-free *E. coli* extract containing the recombinant *Pp*ATaseWT, *Pb*ATaseWT or *Pp*ATaseCH. Empty *E. coli* BL21 (DE3) host cells served as positive control (C). The ATase encoding genes *phlA*, *phlC* and *phlB* of all ATases were overexpressed in soluble form.

4. Alternative ATase Preparations and Storage Types

Lyophilized cells/KPi or PBS. The harvested cells were washed and resuspended in KPi-buffer (50 mM, pH 7.5) or PBS-buffer (137 mM NaCl, 2.7 mM KCl, 10 mM Na_2HPO_4, 1.8 mM KH_2PO_4), shock-frozen in liquid nitrogen and lyophilized. The cells were stored at 4 °C (optional: inert storage under Ar) until further use for biotransformations.

Lyophilized cell-free extract. The harvested cells were suspended in buffer (7 mL buffer to 1 g wet cells; KPi-buffer, 50 mM, pH 7.5) and disrupted to obtain the cell-free extract. The cell-free extract was shock-frozen in liquid nitrogen, lyophilized and stored at 4 °C (optional: inert storage under Ar) until further use for biotransformations.

Cell-free extract. The harvested cells were suspended in buffer (7 mL buffer to 1 g wet cells; KPi-buffer, 50 mM, pH 7.5) and disrupted to obtain the cell-free extract. The cell-free extract was shock-frozen in liquid nitrogen and stored at 4 °C, -20 °C or -80 °C until further use for biotransformations.

Culture media. Protein expression was tested in different culture media: LB-, TB- and YENB-media.

LB-media (1 L): 10 g tryptone (Oxoid™), 7 g NaCl (Roth), 5 g yeast extract (Oxoid™), optional: 1 mM $ZnCl_2$, fill up to 1 L with H_2O, autoclave.

TB-media (1 L): 12 g tryptone (Oxoid™), 24 g yeast extract (Oxoid™), 4 mL glycerol, fill up to 900 mL with H_2O, autoclave. Add 100 mL of a sterile solution of 0.17 M KH_2PO_4, 0.72 M K_2HPO_4.

YENB-media (1 L): 0.8% nutrient broth (Difco™), 0.75% Bacto yeast extract (Difco™), 12 N NaOH (adjust pH 7.5), fill up to 1 L with H_2O, autoclave.

5. Modified procedure to test the impact of bivalent metals

The chloride salt of Ca^{2+}, Mg^{2+}, Zn^{2+}, Cu^{2+}, Co^{2+}, Mn^{2+}, Sr^{2+} or Ni^{2+} (5.0 or 8.0 mM final concentration) was added to the reaction mixture containing ATase and substrate (Figure S9).

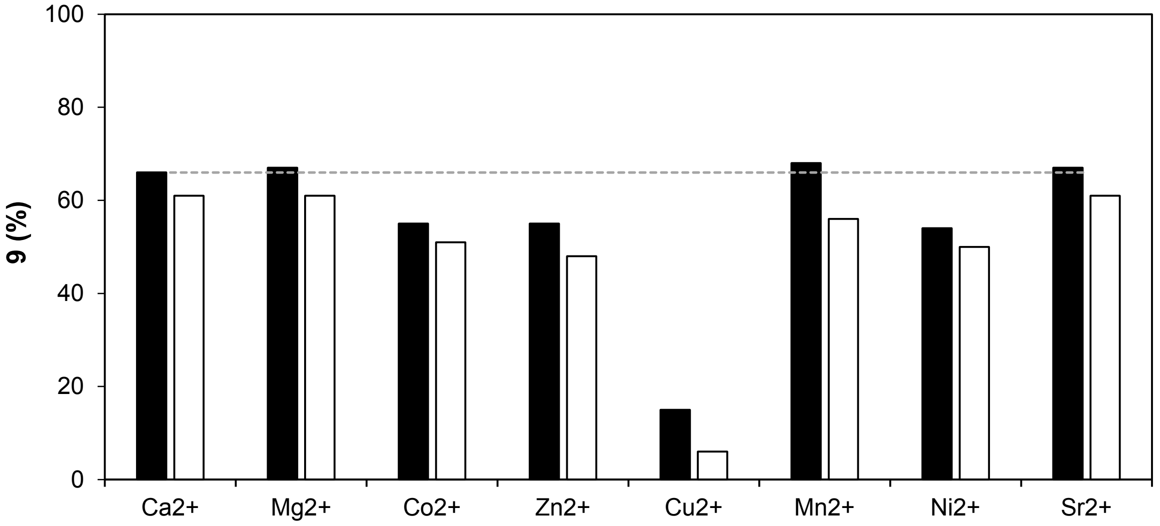

Figure S9. The influence of bivalent metals on the biocatalytic reaction catalyzed by *Pp*ATaseCH. Chloride salts were added to the reaction at different concentrations, *i.e.* 5.0 mM (black columns) or 8.0 mM (red columns). The control reaction was performed in the absence of metal salts (dashed grey line). Assay conditions: Lyophilized cell of *E. coli* extract containing the recombinant *Pp*ATaseCH (20 mg), HEPES-buffer (50 mM, pH 7.5), solution of M^{2+} (5.0 mM or 8.0 mM, prepared in HEPES-buffer using the corresponding metal chloride salt MCl_2), resorcinol (**1b**, 10 mM), DAPG (15 mM), 35 °C, 30 minutes, 750 rpm.

The bioacetylation of **8** with DAPG was performed as described in the manuscript. The influence of various temperatures on expression visibility are shown in Figure S10.

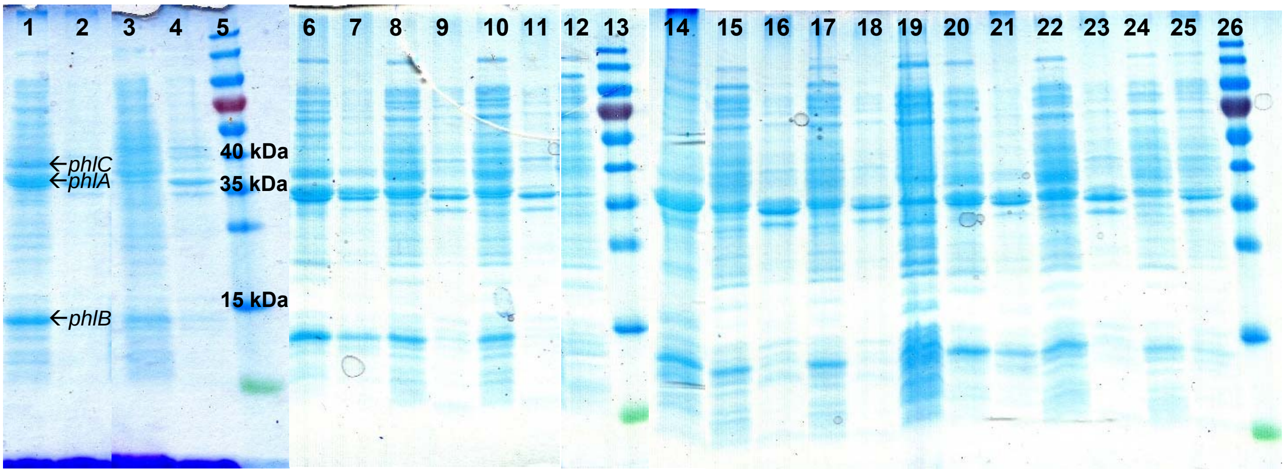

Figure S10. SDS-PAGE of the *Pp*ATaseCH and the *Pp*ATaseCH (pCAS1-construct) in comparison to the *Pp*ATaseWT after expression in the presence or absence of $ZnCl_2$ (1 mM) for 21 h at different temperatures: *Pp*ATaseCH 30 °C, Zn^{2+}, supernatant (lane 1), pellet (lane 2); *Pp*ATaseWT 30 °C, Zn^{2+}, supernatant (lane 3),

pellet (lane 4); PageRuler Prestained Protein Ladder (lane 5, 13, 26); *Pp*ATaseCH 37 °C, Zn^{2+}, supernatant (lane 6), pellet (lane 7); *Pp*ATaseCH 25 °C, Zn^{2+}, supernatant (lane 8), pellet (lane 9); *Pp*ATaseCH 20 °C, Zn^{2+}, supernatant (lane 10), pellet (lane 11); *Pp*ATaseCH-pCAS1 37 °C, Zn^{2+}, supernatant (lane 12), pellet (lane 14); *Pp*ATaseCH-pCAS1 25 °C, Zn^{2+}, supernatant (lane 15), pellet (lane 16); *Pp*ATaseCH-pCAS1 20 °C, Zn^{2+}, supernatant (lane 17), pellet (lane 18); negative control, empty pASK-IBA3 vector (lane 19); *Pp*ATaseCH 37 °C, w/o Zn^{2+}, supernatant (lane 20), pellet (lane 21); *Pp*ATaseCH 25 °C, w/o Zn^{2+}, supernatant (lane 22), pellet (lane 23); *Pp*ATaseCH 20 °C, Zn^{2+}, supernatant (lane 24), pellet (lane 25).

Different water-immiscible (toluene, cyclohexane), moderately water-miscible (MTBE, DIPE, Et_2O, EtOAc), aprotic water-miscible (DMSO, DMF, THF, 1,4-dioxane, acetone, MeCN) and protic water-miscible (MeOH, EtOH, glycerol, ethylene glycole) solvents were tested for the bioacetylation of model substrate **8** either at 5 vol% (Figure S11, black columns) or 20 vol% (Figure S11, grey columns) and the compatibility with *Pp*ATaseCH was determined based on the formation of *C*-acetyl product **9**.

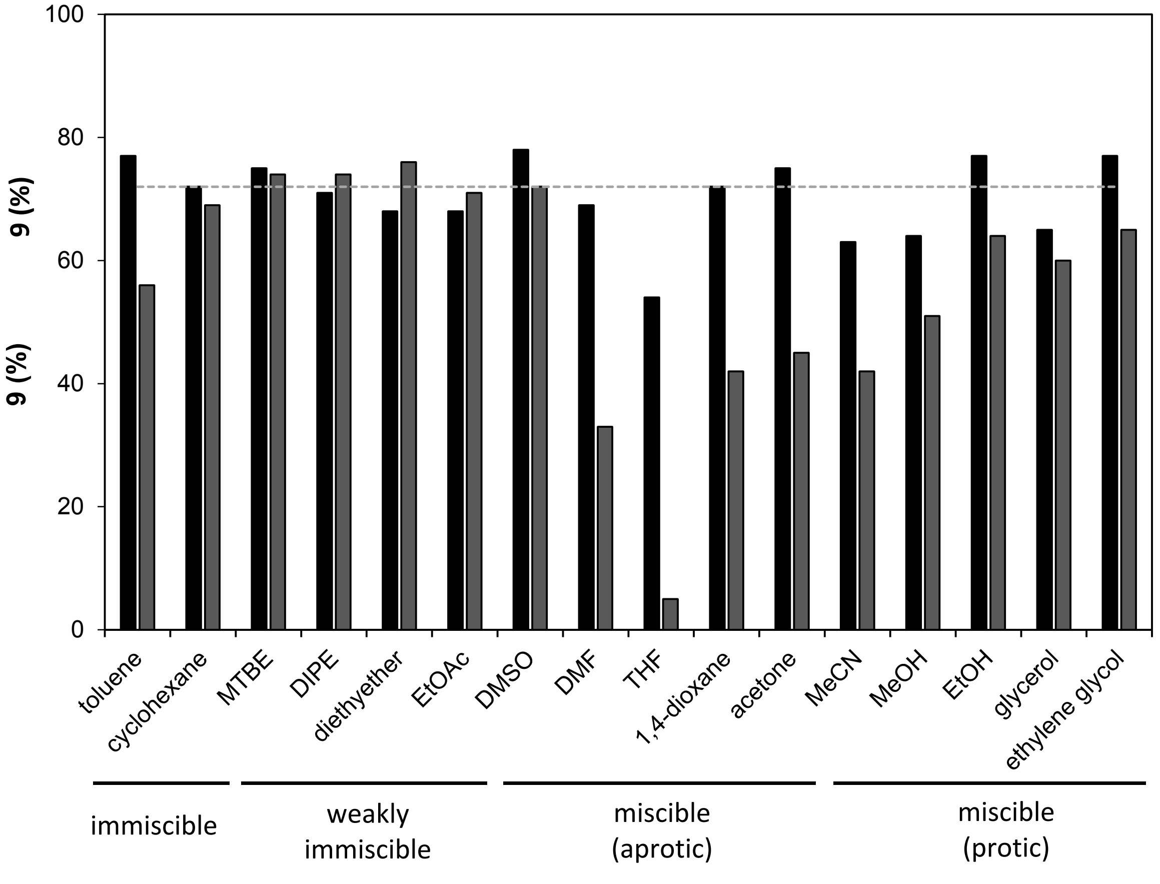

Figure S11. Co-solvent-study for the acetylation of 8 employing *Pp*ATaseCH at 5 vol% (black columns) or 20 vol% (grey columns) of solvent. Assay conditions: Cell-free *E. coli* extract containing the recombinant *Pp*ATaseCH (vol. ≡ to 20 mg lyophilisate), KPi-buffer (50 mM, pH 7.5), resorcinol (**8**, 10 mM), DAPG (15 mM), co-solvent (5 or 20 vol%), 35 °C, 30 minutes, 750 rpm.

6. Modified procedure to test the impact of inhibitors/additives

A small aliquot of cell-free *E. coli* extract containing the recombinant ATase (50 µL) was pretreated with the respective inhibitor/additive for 40 minutes at 28 °C: dithiothreitol (DTT, 0.5 or 2 mM), 2-mercaptoethanol (β-Met, 1 or 2 mM), phenylmethanesulfonyl fluoride (PMSF, 1 mM), iodoacetic acid (IAA, 1 or 2 mM), p-chloromecuribenzoic acid (pCMB, 1 mM), diethylpyrocarbonate (DEPC, 2 or 3 mM), EDTA (5 mM), Triton-X100 (0.5 w/v%), Tween-40 (0.5 w/v%). The residual activity of the pretreated ATase was determined by performing the bioacetylation of 8 with DAPG for 30 minutes at 35 °C as described in manuscript.

Additionally, the effect of phloroglucinol (PG, 10 mM), resorcinol (8, 10 mM), monoacetylphloroglucinol (MAPG, 15 mM), 2,4-dihydroxyphloroglucinol (DAPG, 15 mM) was examined. A solution containing the recombinant ATase (50 µL), KPi-buffer (50 mM, pH 7.5, total volume: 0.5 mL), phloroglucinol (PG, final conc.10 mM) or resorcinol (8, final conc. 10 mM) was incubated for 40 minutes at 28 °C. After this time, the bioacylation was started by addition of DAPG (1.58 mg, 0.0075 mmol, 15 mM final concentration) dissolved in DMSO (50 µL). The final reaction mixture (0.5 mL, 10 vol% DMSO) was shaken for 30 min at 35 °C and 750 rpm in an Eppendorf benchtop shaker. The reaction was aborted by addition of HPLC-grade MeCN (0.45 mL) and vigorous shaking. The precipitated protein was removed by centrifugation (20 min, 14,000 rpm, 18,407 x g) and the supernatant (800 µL) was directly subjected to HPLC for determination of conversions.

In the case of MAPG (final conc. 15 mM) or DAPG (final. Conc. 15 mM) after incubation with enzyme, the bioacylation was started by addition of resorcinol (8, 10 mM final conc.) dissolved in DMSO (50 µL).

7. Synthesis of 2,4-diacetylphloroglucinol (DAPG, 5)

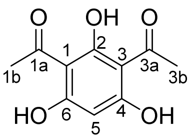

According to a literature procedure,[4] phloroglucinol (500 mg, 4.0 mmol) was dissolved in $BF_3 \cdot 2CH_3COOH$ (2.5 mL, 18.0 mmol) and the resulting mixture was refluxed for 3 h. After cooling the mixture to room temperature, a solution of 0.5 M aqueous KOAc (50 mL) was added dropwise and stirring was continued for further 30 minutes. The crude precipitate was filtered and recrystallized from MeOH/H_2O (1:1) affording 2,4-diacetylphloroglucinol **5** as orange needle-shaped crystals (767 mg, 3.65 mmol, 91 %). R_F = 0.8 ($CHCl_3$/MeOH, 80:20), m.p 143-145 °C (173-174 °C). NMR data is in accordance with literature.[3] ^{1}H-NMR (300 MHz, DMSO-*d6*): δ [ppm] = 2.57 (s, 6 H, 1b-H, 3b-H), 5.85 (s, 1 H, 5-H), 13.19 (s, 2 H, Ar-OH), 16.27 (s, 1 H, Ar-OH). ^{13}C-NMR (75 MHz, DMSO-*d6*): δ_C [ppm] = 32.97 (C-1b), 95.05, 104.0, 169.1, 171.6 (4 × arom. C), 204.0 (C-1a). GC-MS (EI$^+$, 70 eV): *m/z* (%) = 210 [M+] (67), 195 [$C_9H_8O5^+$] (100), 177 [$C_{10}H_9O_3^+$] (64).

References

[1] C. Notredame, D. G. Higgins, J. Heringa, *J. Mol. Biol.* **2000**, *302*, 205-217.

[2] X. Robert, P. Gouet, *Nucleic Acids Res.* **2014**, *42*, W320-W324.

[3] S. Sato, T. Kusakari, T. Suda, T. Kasai, T. Kumazawa, J.-i. Onodera, H. Obara, *Tetrahedron* **2005**, *61*, 9630-9636.

[4] A. V. Bryksin, I. Matsumura, *BioTechniques* **2010**, *48*, 463-465.

[5] D. G. Gibson, L. Young, R.-Y. Chuang, J. C. Venter, C. A. Hutchison, H. O. Smith, *Nat. Meth.* **2009**, *6*, 343-345.
